# Quantitative structure-property relationships for predicting sorption of pharmaceuticals to sewage sludge during waste water treatment processes

**DOI:** 10.1016/j.scitotenv.2016.11.156

**Published:** 2017-02-01

**Authors:** L. Berthod, D.C. Whitley, G. Roberts, A. Sharpe, R. Greenwood, G.A. Mills

**Affiliations:** a AstraZeneca Global Environment, Alderley Park, Macclesfield SK10 4TG, UK; b School of Pharmacy and Biomedical Sciences, University of Portsmouth, St Michael's Building, White Swan Road, Portsmouth, Hampshire PO1 2DT, UK; c School of Biological Sciences, University of Portsmouth, King Henry Building, King Henry I Street, Portsmouth, Hampshire PO1 2DY, UK

**Keywords:** Pharmaceuticals, Sewage sludge, Sorption, Partition coefficient, Quantitative structure-property relationship (QSPR), Artificial neural networks

## Abstract

Understanding the sorption of pharmaceuticals to sewage sludge during waste water treatment processes is important for understanding their environmental fate and in risk assessments. The degree of sorption is defined by the sludge/water partition coefficient (*K*_d_). Experimental *K*_d_ values (*n* = 297) for active pharmaceutical ingredients (*n* = 148) in primary and activated sludge were collected from literature. The compounds were classified by their charge at pH 7.4 (44 uncharged, 60 positively and 28 negatively charged, and 16 zwitterions). Univariate models relating log *K*_d_ to log *K*_*ow*_ for each charge class showed weak correlations (maximum R^2^ = 0.51 for positively charged) with no overall correlation for the combined dataset (R^2^ = 0.04). Weaker correlations were found when relating log *K*_d_ to log *D*_*ow*_. Three sets of molecular descriptors (Molecular Operating Environment, VolSurf and ParaSurf) encoding a range of physico-chemical properties were used to derive multivariate models using stepwise regression, partial least squares and Bayesian artificial neural networks (ANN). The best predictive performance was obtained with ANN, with R^2^ = 0.62–0.69 for these descriptors using the complete dataset. Use of more complex Vsurf and ParaSurf descriptors showed little improvement over Molecular Operating Environment descriptors. The most influential descriptors in the ANN models, identified by automatic relevance determination, highlighted the importance of hydrophobicity, charge and molecular shape effects in these sorbate-sorbent interactions. The heterogeneous nature of the different sewage sludges used to measure *K*_d_ limited the predictability of sorption from physico-chemical properties of the pharmaceuticals alone. Standardization of test materials for the measurement of *K*_d_ would improve comparability of data from different studies, in the long-term leading to better quality environmental risk assessments.

## Highlights

• Understanding sorption of pharmaceuticals to sludge is important in risk assessment.
• Predicting sorption on molecular properties limited by heterogeneous nature of sludge
• Models based only on the partition coefficient gave poor predictive models.
• Non-linear artificial neural network models improve predictability.
• Descriptors that influenced sorbate-sorbent interactions were identified.

## Graphical abstract

**Figure:**
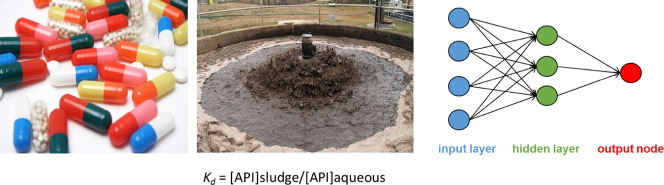

## 1 Introduction

The fate of active pharmaceutical ingredients (APIs) and personal care products in the aquatic environment has been studied over the last twenty years (Daughton, 2016, Kümmerer, 2008, Kümmerer, 2010, Hester and Harrison, 2016). This is due in part to their potential toxicological effects on organisms (Brausch et al., 2012, Corcoran et al., 2010, Vestel et al., 2016). These substances enter the environment through a number of routes, with domestic and hospital waste effluents considered the most important sources (Nikolaou et al., 2007, Monteiro and Boxall, 2010). Some APIs can be removed in waste water treatment plants (WWTPs). However, most WWTPs were not designed for this specific purpose, as historically APIs were unregulated compounds. Many APIs are recalcitrant and are not fully biodegraded during treatment processes. For example propranolol can be extensively removed during treatment, whilst carbamazepine and atenolol have elimination rates below 10% (Miège et al., 2009, Gardner et al., 2012, Gardner et al., 2013). During these processes APIs can be modified to form new products and API metabolites and conjugates produced by secondary detoxification may be degraded back to the parent drug moiety (Petrie et al., 2015). The amount of some APIs entering the environment (e.g. those for the treatment of cardio-vascular disease and diabetes) is predicted to increase in the future due to increasing and ageing populations.

During waste water treatment, APIs can be removed by sorption as well as biodegradation. The degree of partitioning between sludge and aqueous phases in different sections of the treatment process is often used as a key indicator of their environmental fate and in risk assessments for the subsequent disposal of sludge to land (Berthod et al., 2014). The distribution of an API between these two phases is described by the partition coefficient *K*_d_, which is defined as the dimensionless ratio of the equilibrium concentrations (expressed as L kg^− 1^) of the substance in the sludge and aqueous phases:

(1) $$K_{d}={\left[\mathrm{API}\right]}_{\text{sludge}}/{\left[\mathrm{API}\right]}_{\text{aqueous}}$$

Values of *K*_d_ have been reported in different sludge types for various pharmaceutical classes including antibiotics, anticancers, anti-inflammatories, cardiovasculars, central nervous system drugs and hormones (Andersen et al., 2005, Barron et al., 2009, Carballa et al., 2008, Clara et al., 2004, Göbel et al., 2005, Hörsing et al., 2011, Hyland et al., 2012, Jia et al., 2012, Joss et al., 2005, Kim et al., 2010, Lajeunesse et al., 2012, Radjenović et al., 2009, Stasinakis et al., 2013, Stevens-Garmon et al., 2011, Stuer-Lauridsen et al., 2000, Suárez et al., 2008, Ternes et al., 2004, Urase and Kikuta, 2005, Wick et al., 2009, Yan et al., 2014). However, measured *K*_d_ values are not publically available for many prescription and over-the-counter pharmaceuticals.

Typically, four types of sludge are found within a WWTP: primary, activated, secondary and anaerobically digested, and *K*_d_ values have been measured in all of these matrices. Different sludge types have been shown to influence the sorption behaviour of APIs (Berthod et al., 2016). This is to be expected in view of the variation in bulk characteristics (e.g. total suspended solids, total organic carbon and chemical oxygen demand) of sludges across and within different WWTPs (Berthod et al., 2016). In addition, surface properties of the sludge and the local operating pH within the WWTP have been shown to be influential factors governing sorption processes (Hörsing et al., 2011, Ternes et al., 2004).

Normally, sorption behaviour of APIs is measured using a standard test, such as the Organisation for Economic Co-operation and Development (OECD) test 106 (OECD, 2000), OECD test 121 (OECD, 2001) or the US Environmental Protection Agency's Office of Prevention, Pesticides and Toxic Substances (OPPTS) guideline 835.1110 (EPA, 1998). The OECD 106 or OPPTS 835.1110 tests are required by the European Medicines Agency for measuring partition behaviour of substances in sewage sludge in environmental risk assessments, with the former test preferred (EMA, 2015). It involves experiments using different aqueous concentrations of sludge and the slope of the linear section of the resultant isotherm is used to estimate *K*_d_. The guideline dictates the use of activated sewage sludge as test medium, even though this matrix may not be representative of all the partitioning mechanisms occurring in a WWTP. This procedure is time-consuming and labour-intensive, and alternative experimental approaches for measuring *K*_d_ have been investigated, for example using solid-phase extraction techniques (Berthod et al., 2014).

The use of predictive mathematical models to estimate *K*_d_ may provide faster, lower-cost alternatives to laboratory-based methods. This is attractive for preliminary assessment of environmental fate and associated risks in early development of candidate APIs. Identification of suitable models for the prediction of *K*_d_ is challenging as APIs occupy specific regions of physico-chemical property space (such as those identified by the Lipinski ‘rule of 5’ (Lipinski et al., 1997)) in order to be effective therapeutic agents. Furthermore, many drugs are ionisable (e.g. acids, bases and zwitterions), which adds complexity compared with neutral compounds, where predictions may be made based solely on the octanol/water partition coefficient, log *K*_*ow*_.

Typically, early models were adapted from work on soils and focussed on hydrophobicity as the major mechanism governing sorption, leading to linear relationships of the form log *K*_d_ = *a* + *b* log *K*_*ow*_. Often, an organic carbon normalised version of *K*_d_, the organic carbon partition coefficient, *K*_*OC*_, which is obtained from *K*_d_ = *K*_*OC*_ × *f*_*OC*_, where *f*_*OC*_ is the fraction of organic carbon in the sludge, was used (Schwarzenbach et al., 2002). This simple approach has been used to identify APIs with low (log *K*_*OW*_ < 2.5), medium (2.5 < log *K*_*OW*_ < 4.0) and high sorption potential (log *K*_*OW*_ > 4.0) (Rogers, 1996). APIs with high log *K*_*OC*_ values (> 4.0) require further terrestrial risk assessment (EMEA, 2006) as disposal of sludge to agricultural land involves potential risks associated with their leaching and movement into crops destined for livestock or human consumption.

These single-parameter models often fail to predict accurately the *K*_d_ value for ionisable APIs, where hydrophobicity is no longer the sole mechanism of sorption. Subsequent workers have sought to address this by developing models that include other properties, such as the Abraham linear free energy descriptors, in a multivariate quantitative structure-property relationship (QSPR) approach (Abraham et al., 2004, Faria and Young, 2010). Similar multivariate descriptor-based models have been derived for sorption of non-ionic pesticides to soils (Gramatica et al., 2000).

In effluents, the pH across different WWTPs can range typically from 7 to 8 (Berthod et al., 2016), which affects the charge state of ionisable APIs. Consequently, models have also been developed for APIs in different ion classes (Dobbs et al., 1989, Franco et al., 2009, Franco et al., 2013, Franco and Trapp, 2008, Guo et al., 2004, Sabljic et al., 1995). Recent work by Sathyamoorthy and Ramsburg (2013) derived separate multivariate models to predict *K*_d_ values (at the experimental pH used for the measurements) for uncharged, and positively and negatively charged APIs, and combined models for their whole dataset.

Here we extend this approach using a larger dataset of *K*_d_ values for ionisable APIs measured in activated and primary sewage sludge. Earlier work showed these two sludge types exhibit similar sorption behaviour for such compounds (Berthod et al., 2016). Combining activated and primary sludge types increased the number of data points available for model derivation. In addition to a collection of standard molecular properties, we have also used other descriptor sets (VolSurf and ParaSurf) to develop linear models using partial least squares (PLS). Bayesian regularized artificial neural networks (ANNs) were used with these descriptor sets to derive non-linear models. ANNs have been used to obtain predictive models of complex environmental data sets (Barron et al., 2009, Miller et al., 2016). The ANNs are trained by adjusting their parameters to minimize an error, or cost, function on a subset of training data. Their generalization ability is assessed by their performance on a smaller test set that is excluded from the training procedure. This work aimed to provide models with improved predictive performance to aid in understanding the fate of APIs within water treatment processes, and identify key physico-chemical properties of ionisable APIs that potentially influence sorption processes to sewage sludge.

## 2 Methods

### 2.1 Sorption data

Published *K*_d_ values were found by searching standard scientific bibliographical databases, and from AstraZeneca environmental risk data (AstraZeneca, 2016), AstraZeneca in-house reports and new experimental values were obtained using a solid-phase extraction method (Berthod et al., 2014). The *K*_d_ values were restricted to primary and activated sludge types in conventional activated sludge (CAS) WWTPs (Berthod et al., 2016). This resulted in a dataset of 297 experimental *K*_d_ values for 148 APIs and major metabolites (Table S1). Sorbate-sorbent interactions are influenced strongly by the charge of the dominant species of an API at the pH of the environmental medium (Sathyamoorthy and Ramsburg, 2013). The experimental pH was reported for 142 (48%) of the *K*_d_ values and ranged from 6.30 to 7.74 with a mean value of pH 7.03.

### 2.2 Chemical structures

Structures for the above APIs were downloaded in structure data file (SDF) format from the chemicalize.org website (Chemaxon, 2016). The protomer at the experimental pH was chosen by visual inspection of the graphs of concentration versus pH for the microspecies on chemicalize.org. The majority of compounds had the same major species in the range pH 6–8. Of the compounds where more than one species was present at the experimental pH, there were only three (cimetidine, irbesartan and nefazodone) where the species at the experimental pH clearly differed from the major species at pH 7.4. Apart from these, the major species at pH 7.4 were used. The dataset was divided according to charge state of the APIs at pH 7.4: uncharged (*n* = 44), positively (*n* = 60) and negatively (*n* = 28) charged compounds, together with a small number of zwitterionic (*n* = 16) substances. Energy minimized, three-dimensional conformations were generated from the SDF structures using the Molecular Operating Environment (MOE) (MOE, 2012). Atomic partial charges were assigned using the partial equalization of orbital electronegativities (PEOE) method (Gasteiger and Marsili, 1980). Canonical SMILES (Weininger, 1988, Weininger et al., 1989) for the APIs were generated by MOE from the SDF structures (Table S2).

### 2.3 Univariate models predicting log *K*_d_

Linear regression models for log *K*_d_ in terms of log *K*_*ow*_ and the octanol/water distribution coefficient log *D*_*ow*_ at pH 7.4 were derived using Minitab (Minitab, 2016). Log *D*_*ow*_ takes into account the ionised proportion of a molecule at a given pH and depends on log *K*_*ow*_ and the p*K*_*a*_ of the ionisable functional groups. The log *K*_*ow*_ values for the parent compounds reported on the chemicalize.org website, calculated by a modified version of the method of Viswanadhan et al. (1989), were included in the downloaded SDF files (Table S3). Log *D*_*ow*_ values at pH 7.4 were obtained from ChemSpider (www.chemspider.com). Models were calculated for each of the four charge states, and for the combined dataset, and their performance assessed by the coefficient of determination, R^2^.

### 2.4 Selection of molecular descriptors, training and test sets for multivariate models

A set of 23 molecular descriptors characterizing properties potentially involved in sorption (hydrophobicity, size, shape and polarity) were calculated with MOE (Table S4). These were supplemented with log *K*_*ow*_ and log *D*_*ow*_ at pH 7.4 to give a total of 25 descriptors. A set of 76 Vsurf descriptors were calculated (Table S5), using the MOE implementation of the VolSurf descriptors (Cruciani et al., 2000a, Cruciani et al., 2000b) derived from volumes and surfaces of three-dimensional molecular interaction fields. A set of 81 ParaSurf descriptors (Lin and Clark, 2005) encoding electronic molecular surface properties (molecular electrostatic potential, local electron affinity, local ionization energy and local polarizability), was calculated based on semi-empirical wave functions computed with EMPIRE (Hennemann and Clark, 2014). For each descriptor set, variables with a coefficient of variation (CV, the standard deviation divided by the mean) below 0.05 were removed and unsupervised forward selection (UFS) was used to remove variables with high multiple correlation (Whitley et al., 2000). UFS selects a set of variables with a minimal degree of multiple correlations. Starting with the two variables with smallest pairwise correlation coefficient, additional variables are added iteratively, with the variable with the smallest multiple correlation coefficient with those already chosen added at each stage. In order to compare the performance of the different descriptor sets and statistical methods, the dataset was divided randomly into training (*n* = 237) and test (*n* = 60) sets, and to investigate the dependence of the results on the choice of test set, the model building was repeated for five independent choices of the test set.

### 2.5 Linear multivariate models predicting log *K*_d_

Stepwise linear regression models were derived using Minitab (Minitab, 2016) for the four charge states, and values of adjusted and predicted R^2^ (R^2^_adj_ and R^2^_pred_) calculated. Compounds with high leverage were removed and models recomputed on the reduced dataset. Similarly, PLS models were derived using Minitab. PLS models were also computed using the five training sets for the combined charge classes. The leave-one-out cross validated R^2^ (R^2^_cv_) was used to select the number of components, and mean unsigned errors (MUE) and R^2^ values were calculated for the training and test sets.

### 2.6 Artificial neural network models predicting log *K*_d_

The ANNs were feed-forward, multi-layer perceptrons with three layers: an input layer containing one node for each descriptor, a hidden layer of nodes using hyperbolic tangent activation functions and an output layer consisting of a single node representing the predicted value of log *K*_d_. The number of nodes in the hidden layer was varied from 2 to 5. The ANN topology is shown in Fig. S1. A single hidden layer was used as such networks are able to approximate arbitrary smooth functions (Blum and Li, 1991), and the number of nodes in the hidden layer was kept low to reduce the chance of over-fitting the relatively small data set.

ANNs were trained using Mackay's ‘evidence’ framework (Mackay, 1992), based on Bayesian techniques. ANNs are parameterized by a set of network weights and conventional training finds a single, optimal set of weights by using error back-propagation to minimize an error function. The Bayesian approach deals instead with probability distributions of network weights. From a prior distribution of weights and an observed dataset, a posterior weight distribution is inferred, and the maximum of the posterior distribution is chosen as the optimal set of weights. For data with a Gaussian noise model and a prior distribution penalizing large weights, this amounts to minimizing a sum-of-squares error function with a weight decay regularization term (Bishop, 1995). The evidence framework uses a maximum likelihood estimate of the hyperparameters controlling the weight decay and the variance of the noise. The most important descriptors were identified using automatic relevance determination (ARD) (Burden et al., 2000, MacKay, 1996), which applies a separate regularization parameter to the weights associated with each input node. Inputs with small weight decay parameters have larger weights, hence more influence on the network output, and this provides a ranking of the relevance of each descriptor.

ANN calculations were done using the Netlab toolkit (Nabney, 2002, Nabney, 2004) in Matlab (Matlab, 2016). For each choice of the number of hidden nodes, 100 networks were trained and the MUE and R^2^ averaged over the best (measured by log evidence) ten networks for both training and test sets were reported. The analysis was repeated for the MOE, Vsurf and ParaSurf descriptor sets. These sets were treated independently to enable comparisons between them and to produce QSPR models available for end-users dependent only on a single commercial package.

## 3 Results and discussion

Some APIs had several reported *K*_d_ values, often obtained under different experimental conditions and in different sludge types. For example, there were 12 values (log *K*_d_ = 0.08–2.50) for carbamazepine, 10 for diclofenac (log *K*_d_ = 1.20–2.66) and 10 for trimethoprim (log *K*_d_ = 1.88–2.63). For APIs for which two or more experimental *K*_d_ values were available, the mean and median ranges of log *K*_d_ for the whole dataset were 0.91 and 0.82, respectively. To obtain as large a dataset as possible to develop the QSPR models, all the available *K*_d_ values were used, rather than taking a mean value for each API. This added inherent variability to the dataset. Sorption of APIs to sewage sludge is complex, involving factors such as hydrophobic, hydrogen-bonding and charge-related interactions. The charge state of the APIs is therefore important in understanding such sorbate-sorbent interactions. Hence, for modelling purposes, the *K*_d_ values for the APIs were divided into four groups according to the charge state of the dominant species at the experimental pH. The number of available *K*_d_ values for zwitterionic APIs was considerably lower (*n* = 24), making robust predictive models more difficult to obtain.

### 3.1 Univariate models predicting log *K*_d_

**Fig. 1:**
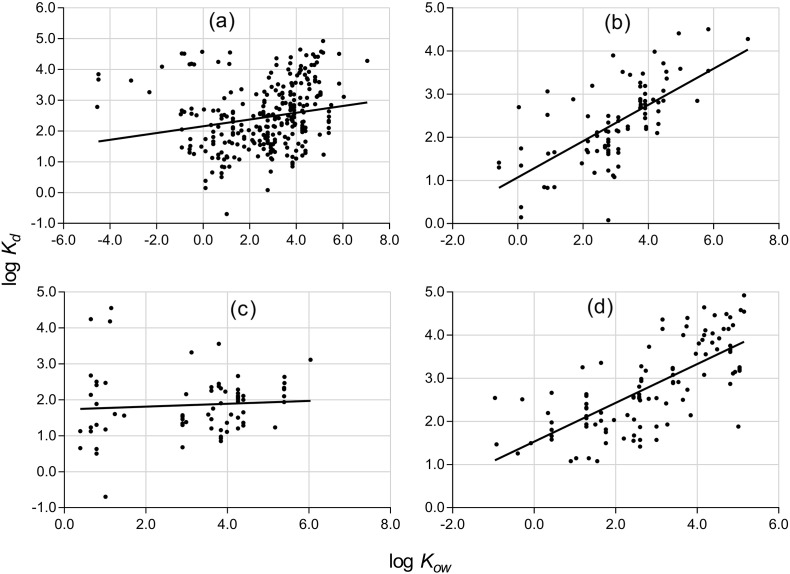
Plots of reported log *K*_d_ values (L kg^− 1^) against log *K*_*ow*_ for APIs: (a) complete dataset (*n* = 297), linear regression equation log *K*_d_ = 0.11 log *K*_*ow*_ + 2.15, R^2^ = 0.04; (b) uncharged compounds (*n* = 92), linear regression equation log *K*_d_ = 0.42 log *K*_*ow*_ + 1.07, R^2^ = 0.46; (c) negatively charged compounds (*n* = 75), linear regression equation log *K*_d_ = 0.04 log *K*_*ow*_ + 1.73, R^2^ = 0.01; (d) positively charged compounds (*n* = 105), linear regression equation log *K*_d_ = 0.45 log *K*_*ow*_ + 1.53, R^2^ = 0.51. Zwitterionic compounds (*n* = 24) omitted. Ceftadizime (log *K*_d_ = − 4.55) omitted from the negatively charged dataset.

Several studies have used the octanol/water partition coefficient as the sole predictor of sorption behaviour of compounds to soil, sediment and sludge (Dobbs et al., 1989, Franco and Trapp, 2008, Guo et al., 2004, Huuskonen, 2003, Sabljic et al., 1995). This approach was used in earlier studies to predict the sorption behaviour of uncharged APIs (*n* ≤ 19) in sewage treatment processes (Huuskonen, 2003, Hyland et al., 2012, Stevens-Garmon et al., 2011, Sabljic et al., 1995). More recent work (Sathyamoorthy and Ramsburg, 2013) using a larger dataset investigated the ability of log *K*_*ow*_ to predict sorption of positively charged (108 values for 32 APIs), negatively charged (65 values for 16 APIs) and uncharged (44 values for 19 APIs) compounds. A similar approach was adopted to compare the behaviour of our dataset. Linear regressions of log *K*_d_ on log *K*_*ow*_ for the complete dataset and for the subsets of uncharged, and negatively and positively charged APIs are shown in Fig. 1. Results for zwitterionic compounds, identified as those with non-zero atomic partial charges but zero total charge at the experimental pH (or pH 7.4 when the experimental pH was not reported) and regressions of log *K*_d_ on log *D*_*ow*_ at pH 7.4 are shown in Table S6. Several APIs had large leverage values in the models (Table S7). New models were calculated with these compounds removed (Table S6).

Significant correlations between log *K*_d_ and log *K*_*ow*_ were found only for uncharged and positively charged compounds, in agreement with the work of Sathyamoorthy and Ramsburg (2013). However, our dataset gave improved R^2^ values for uncharged (R^2^ = 0.47) and positively charged (R^2^ = 0.51) compounds compared to the values of R^2^ = 0.40 and R^2^ = 0.36, respectively, reported by Sathyamoorthy and Ramsburg (2013). Interestingly, the slopes of the regression lines for uncharged (0.42) and positively charged (0.45) compounds were similar, indicating a similar dependence of log *K*_d_ on log *K*_*ow*_. Removal of outliers produced only marginal improvements in correlations (Table S6). Correlations between log *K*_d_ and log *D*_*ow*_ (Table S6) followed the same pattern, but with smaller R^2^ values for uncharged (R^2^ = 0.36) and positively charged (R^2^ = 0.37) APIs compared to those for the log *K*_*ow*_ models. For negatively charged compounds and zwitterionic APIs, negligible correlations between log *K*_d_ and both log *K*_*ow*_ and log *D*_*ow*_ were found. These results for log *D*_*ow*_ models differ from Sathyamoorthy and Ramsburg (2013), who found an improved R^2^ value for negatively charged and uncharged compounds, but a weaker R^2^ value for positively charged APIs. Precise reasons for the observed differences between the results of the two studies are difficult to assign, but are likely to be due to differences in the datasets used. As log *K*_*ow*_ and log *D*_*ow*_ both measure hydrophobicity, these results suggest that hydrophobic interactions play a large role for uncharged and positively charge compounds, while other types of interaction (e.g. electrostatic) must be included to obtain predictive models for negatively charged compounds. Overall these findings show that univariate equations based on the partition coefficient yield weakly predictive models, and indicate that additional predictors are required to account for non-hydrophobic interactions.

### 3.2 Linear multivariate models predicting log *K*_d_

Very many descriptors are available for the development of QSPR models, in areas such as drug discovery and toxicity prediction (Todeschini and Consonni, 2009). We investigated three sets of descriptors (MOE, Vsurf and ParaSurf) incorporating properties and interactions likely to be involved in sorption of APIs to sewage sludge. The MOE descriptors include standard one- and two-dimensional physico-chemical properties while the Vsurf and ParaSurf descriptor sets encode more complex three-dimensional structures and properties. Multivariate models for the prediction of sorption of organic chemicals have been explored previously (Doucette, 2003) but these used a more restricted collection of descriptors.

For each descriptor set and charge class, removal of variables with CV < 0.05 and variables with high multiple correlation identified by UFS, reduced the datasets to the sizes shown in Table S8. Results for stepwise regression models for separate charge classes are shown in Table 1. The inclusion of additional descriptors improved the R^2^ values compared with those obtained in univariate models (Fig. 1). For the uncharged APIs, R^2^ increased from 0.46 to between 0.51 and 0.58 for the three descriptor sets. A similar improvement was observed for the positively charged APIs, with R^2^ increasing from 0.51 to between 0.66 and 0.79. The most noticeable increase in R^2^ occurred for the negatively charged APIs, from effectively zero to between 0.57 and 0.63. Likewise for the smaller set of zwitterions, R^2^ increased to between 0.39 and 0.42. However, in some cases this improvement was at the expense of models containing a large number of variables. Overall, the added complexity of the Vsurf and ParaSurf descriptors gave no clear improvement for any of the charge classes.

As expected, for the uncharged and positively charged APIs, the most significant term in the MOE models was log *K*_*ow*_, with smaller contributions from descriptors encoding charge and shape effects. The model for negatively charged APIs included terms for *a_acc* and *a_aro*, with a small contribution from *vsa_hyd* (see Supplementary material for definitions of descriptors). The individual contributions to the Vsurf and ParaSurf models are more difficult to interpret.

To assess the influence of outliers, high leverage compounds identified by Minitab were removed and models retrained (Tables S9–S11). There was little overlap in the outliers removed for the different descriptor sets, and in most cases only minimal improvement in predicted R^2^ was observed. For example, the largest improvement in R^2^_pred_ for the MOE descriptors, from 0.69 to 0.74, occurred for the positively charged compounds, while for the Vsurf descriptors, the biggest increase was from 0.39 to 0.48 for the uncharged APIs. The ParaSurf descriptors produced the largest change in R^2^_pred_, from 0.58 to 0.71 for the positively charged APIs. In several cases, the variables included in the models changed considerably when outliers were removed (see Supplementary material for examples). This sensitivity is likely due to the diversity of *K*_d_ measurements available in the literature used to compile the dataset. This effect is often observed when modelling environmental data obtained from multiple sources (Miller et al., 2016). There are few published multivariate models for comparison, apart from the work of Sathyamoorthy and Ramsburg (2013) based on 15 molecular properties (similar to the MOE descriptors) relating to sorption processes. Their dataset produced better models for the uncharged (R^2^_pred_ = 0.65) and negatively charged (R^2^_pred_ = 0.56) APIs, but a poorer model for the positively charged compounds (R^2^_pred_ = 0.49).

To investigate the performance of linear models including all the descriptors, PLS models were derived for each charge class, with the results shown in Table 2. Apart from the R^2^ value (0.67 versus 0.58) for the ParaSurf descriptors with the uncharged compounds, the results were poorer than those obtained with stepwise regression (Table 1). Removal of outliers did not improve performance of the models (data not shown).

The values for the individual charge classes were combined to allow models to be derived from a larger single dataset. The dataset was divided into training and test sets to provide an estimate of the predictive power of the models. Results for PLS models with five random choices of test set are shown in Table 3.

The results showed little variation between different test sets, suggesting a degree of uniformity across the entire dataset. Coefficients of determination, R^2^, were poor, but average MUEs for each set of descriptors were below 0.59 (training set) and 0.63 (test set), which was less than the variation of many log *K*_d_ values reported for the same API (Table S1). Values of R^2^_test_ were slightly less than R^2^_train_ for the Vsurf and ParaSurf descriptors, suggesting that these models may be able to predict log *K*_d_ well for other APIs. Overall, there was little difference in predictability between the three descriptor sets, using 5–8 PLS components. The nearest comparison with the MOE descriptors is that of Sathyamoorthy and Ramsburg (2013) who derived a six variable model, achieving R^2^ = 0.59, R^2^_pred_ = 0.56.

Tables S12–S14 show the complete list of MOE, Vsurf and ParaSurf descriptors ordered by their loadings on the first three PLS components, averaged over the five choices of test set. The higher the order the more influential the descriptor on the relevant component, and the effect of each component in sorbate-sorbent interactions may be interpreted from the highest ranked descriptors. For the MOE descriptors, variables with the six highest loadings on the first PLS component were hydrophobic properties (a_hyd, vsa_hyd, VSA, log *D*_*ow*_, log *K*_*ow*_, rings); for the second component the highest loadings were for charge and size effects (PC-, a_acc, vsa_pol, a_don, Weight, VSA); the third component was influenced by a combination of these properties (dipole, log *D*_*ow*_, log *K*_*ow*_, a_base, rgyr, vsa_pol). For Vsurf descriptors, variables with the highest loadings on the first component were capacity factor (CW) and minimum hydrophilic energy (EWmin), both measures of hydrophilic effects; for component two, the effects of charge and size were dominant (polar volume (Wp), surface globularity (G), interaction field volume (V), hydrophobic volume (D), H-bond donor capacity (HB)); for component three a mixture of effects were seen (capacity factor (CW), interaction field volume(V), surface rugosity (R), D, h-bond donor capacity (HB), EWmin). Thus the components have similar interpretations to those observed with the MOE descriptors. With ParaSurf, charged surface effects were evident; component one was influenced by local electron affinity (EAL), molecular electrostatic potential (MEP), electric field normal to the surface (FN); component two by local polarizability (POL) and hardness (HARD) and component three by FN, MEP, EAL, electronegativity (ENEG) and local ionization energy (IEL).

These linear multivariate models performed better than the single variable log *K*_*ow*_ models, however, their overall ability to predict the fate of APIs in WWTP remained weak, thereby limiting their usefulness in environmental risk assessments.

### 3.3 Artificial neural network models predicting log *K*_d_

Non-linear models are likely to provide improved predictive power compared with multi-linear approaches. ANNs were used as they provide a method for deriving non-linear models that do not require the form of non-linearity to be known in advance, which is the case in interactions between APIs and sewage sludge.

To reduce the number of parameters in the ANNs, the number of input variables was reduced by applying UFS, resulting in sets of 23, 37 and 41 variables for the MOE, Vsurf and ParaSurf descriptors, respectively.

Networks with 2–5 hidden nodes were investigated using the same training and test sets as those used for the PLS analyses. For each combination of test set and number of hidden nodes, 100 ANNs were trained and MUE and R^2^ values calculated for the best 10 networks identified by Mackay's evidence procedure (Mackay, 1992). This number of ANNs covered a range of local minima of the error function, and little variation between the performances on the different validation sets was observed. This suggested that a reasonable sampling of local minima was achieved. The best test set predictions were obtained for ANNs with two hidden nodes (Tables S15–S17 and Figs. S2–S4). Mean outputs of a committee of the best 10 two-hidden unit networks were calculated for each descriptor set (Table 4). These results improved on the corresponding PLS model performance (Table 3), with the mean R^2^_test_ value increased from 0.45 to 0.58 (MOE), 0.41 to 0.46 (Vsurf) and 0.46 to 0.53 (ParaSurf), while the mean MUE_test_ value fell from 0.62 to 0.54 (MOE), 0.63 to 0.62 (Vsurf) and 0.61 to 0.56 (ParaSurf). The best predictive ANN performance was for the MOE descriptors, followed by the ParaSurf and Vsurf descriptors. The MOE descriptors also had the greatest improvement over the PLS results. The values of R^2^_train_ obtained in Table 4 were lower than those of Barron et al. (2009), who, using a smaller data set (30 compounds), achieved values of R^2^_train_ > 0.88. Their improved results are most likely due to the use of a single sludge for measuring *K*_d_ values, rather than the diverse range of test matrices that were used to generate the dataset in this study. The lower R^2^ values are probably more realistic measures of the predictive ability of these models in practice.

MOE descriptors included hydrophobic (vsa_hyd, log *K*_*ow*_), charge (PC +), hydrogen bond donor/acceptor (a_acc, a_don), polarizability (vsa_pol) and size/shape (rgyr, Weight) properties. Vsurf descriptors were hydrophobic/hydrophilic (EWmin, CW, ID, IW, HL), hydrogen bond donor capacity (HB) and shape (R, G) effects. ParaSurf descriptors included polar (polarizability, POL), electrostatic (MEP, FN) and electronic (HARD, EAL) properties. The diversity of identified descriptors reflects the complexity of the interactions between APIs and sewage sludge. In summary, these were dominated by the effects of hydrophobicity, charge and molecular shape, in agreement with the findings of Barron et al. (2009) and Sathyamoorthy and Ramsburg (2013).

The ten most relevant descriptors identified by ARD in each set are listed in Table 5 (complete list in Table S18).

Examination of the dependence of the network outputs on these variables showed mostly monotonic behaviour with only weak non-linearity. In the few cases where strong non-linearities were observed, this was attributable to the ANNs attempting to fit extreme property values for isolated APIs (data not shown).

All MOE descriptors in Table 5 appeared in the top ten entries for one of the PLS components in Table S12. There was also good correspondence for the Vsurf descriptors, apart from ID7, ID8 (ranked 12 and 13 in component 2) and IW5 (ranked 23 in component 3). The ParaSurf descriptors were more mixed. As the same MOE descriptors were consistently identified as influential by the two multivariate methods, this gives increased confidence that these properties are important in sorbent-sorbate interactions for the APIs.

**Fig. 2:**
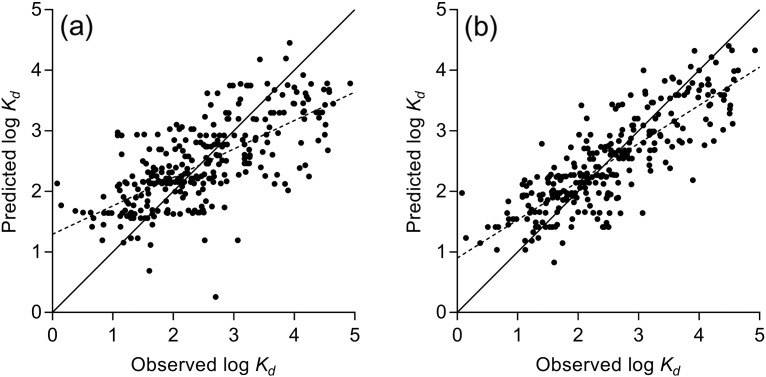
Predicted log *K*_d_ against observed log *K*_d_ for MOE descriptors trained on entire dataset for (a) PLS (R^2^ = 0.47, MUE = 0.58), (b) ANN (R^2^ = 0.64, MUE = 0.47). Data point for zibotentan (observed log *K*_d_ = − 0.699) omitted for clarity.

Having assessed the likely generalization errors, final production PLS and ANN models for each set of descriptors were trained using the entire dataset, following the same protocols. Fig. 2 shows the performance of these models for the MOE descriptors (Vsurf and ParaSurf plots in Figs. S5 and S6, respectively). The R^2^ values were 0.47 (MOE), 0.52 (Vsurf) and 0.56 (ParaSurf) for PLS, and 0.64 (MOE), 0.62 (Vsurf) and 0.69 (ParaSurf) for ANN. As expected, ANNs gave improved predictions. For practical purposes, there were only small differences in performance between the three descriptor sets, and as MOE descriptors are easier to interpret in terms of sorption processes, these are recommended for use by end-users involved in risk assessment studies. The Vsurf and ParaSurf descriptors have not, to our knowledge, been used previously in such environmental applications, but their additional complexity was shown to add little to predictive ability, so their use may not be warranted. To enable application of the MOE model by end users, the Matlab code for prediction of log *K*_d_ values is provided in the Supplementary Material.

## 4 Conclusions

Measured *K*_d_ values reported in the literature show a high level of diversity (e.g. ~ 2.5 log units for carbamazapine), due to the sorption test methods used and composition of the sewage sludge test matrix. As APIs need to act as therapeutic agents, their physico-chemical properties were not uniformly distributed, and there was a predominance of acidic and neutral compounds. This added an inhomogeneity to the dataset used for modelling. Our results showed that hydrophobicity (log *K*_*ow*_) was inadequate as a predictor of sorption of APIs to sewage sludge. Use of multivariate models incorporating additional molecular properties related to the range of sorbate-sorbent interactions, showed the importance of including charge and volume terms. Non-linear ANN models showed improved predictive power over multi-linear PLS models. These models produced MUE_test_ values of 0.61–0.63 (PLS) and 0.54–0.62 (ANN), similar to the variation across the experimental log *K*_d_ measurements, but they failed to produce test-set R^2^ values greater than ~ 0.6. Improved models may be derived for the individual ion classes, but for the non-linear methods this would require larger datasets for training. The best fitting models using the complete dataset were obtained with ANN (R^2^ = 0.62–0.69 for the three descriptor sets) and were a considerable improvement over the single variable log *K*_*ow*_ models.

The modest predictive power of the models developed here may be due in part to the range of sludges with different characteristics (e.g. pH, ionic strength, organic carbon content and oxidation/reduction potential) used to measure *K*_d_. Often these parameters are not determined in sorption tests. This was shown by Barron et al. (2009) who achieved higher R^2^_train_ values when restricting to a single sludge test matrix for a smaller set of 30 compounds. One solution to overcome this problem may be to establish a range of standardized sludges found in the major types of WWTP, on which sorption experiments could be undertaken. A similar approach is taken in soil studies, where reference materials with known properties are available commercially for leaching, degradation and fate measurements of pollutants in long-term ecological studies. This could enable improved comparability between sorption studies undertaken in different laboratories and may lead to the availability of larger, more consistent datasets for use in regulatory environmental risk assessments. This would aid the development of improved QSPRs, which could potentially be used to identify those APIs requiring a terrestrial risk assessment, rather than having to undertake standard laboratory measurements.

## Figures and Tables

**Table 1:** Stepwise regression models for MOE, Vsurf and ParaSurf descriptors, individual charge classes.

Compounds	n	Descriptors	Variables	S	R^2^	R^2^_adj_	R^2^_pred_
Uncharged	92	MOE	3	0.65	0.51	0.50	0.46
		Vsurf	6	0.67	0.51	0.48	0.39
		ParaSurf	7	0.63	0.58	0.54	0.49
Positively charged	105	MOE	8	0.50	0.77	0.75	0.69
		Vsurf	12	0.48	0.79	0.76	0.71
		ParaSurf	7	0.60	0.66	0.64	0.58
Negatively charged	76	MOE	5	0.59	0.57	0.53	0.47
		Vsurf	9	0.56	0.63	0.58	0.50
		ParaSurf	8	0.59	0.59	0.54	0.46
Zwitterions	24	MOE	3	0.79	0.39	0.30	0.17
		Vsurf	2	0.72	0.46	0.41	0.32
		ParaSurf	3	0.77	0.42	0.33	0.27

n = number of *K*_d_ values; S = standard deviation of the error; R^2^_adj_ = adjusted R^2^; R^2^_pred_ = predicted R^2^.

**Table 2:** Partial least squares models for the MOE, Vsurf and ParaSurf descriptors, individual charge classes.

Compounds	n	Descriptors	Variables	Components	R^2^	R^2^_cv_
Uncharged	92	MOE	19	1	0.41	0.35
		Vsurf	39	2	0.45	0.28
		ParaSurf	41	8	0.67	0.33
Positively charged	105	MOE	21	4	0.69	0.62
		Vsurf	43	6	0.78	0.56
		ParaSurf	47	4	0.68	0.51
Negatively charged	76	MOE	18	4	0.57	0.41
		Vsurf	27	1	0.20	0.01
		ParaSurf	25	1	0.28	0.00
Zwitterions	24	MOE	15	3	0.49	0.00
		Vsurf	15	2	0.56	0.01
		ParaSurf	15	2	0.39	0.16

n = number of *K*_d_ values; R^2^_cv_ = leave-one-out cross-validated R^2^.

**Table 3:** Performance of PLS models derived using the combined log *K*_d_ values with five random choices of training (*n* = 237) and test (*n* = 60) sets, for the MOE, Vsurf and ParaSurf descriptors.

Descriptors	Test Set	Components	Variance	MUE_train_	MUE_test_	R^2^_train_	R^2^_cv_	R^2^_test_
MOE	1	8	0.87	0.58	0.62	0.46	0.33	0.50
	2	6	0.81	0.58	0.68	0.47	0.37	0.36
	3	8	0.88	0.59	0.60	0.46	0.34	0.48
	4	5	0.74	0.59	0.62	0.41	0.31	0.50
	5	6	0.82	0.60	0.57	0.44	0.34	0.43
	Mean		0.82	0.59	0.62	0.45	0.34	0.45
	Sdev		0.06	0.01	0.04	0.02	0.02	0.06
Vsurf	1	7	0.71	0.56	0.61	0.49	0.33	0.50
	2	8	0.76	0.52	0.68	0.55	0.41	0.27
	3	6	0.64	0.56	0.64	0.52	0.36	0.37
	4	7	0.67	0.53	0.72	0.53	0.38	0.33
	5	5	0.61	0.59	0.51	0.45	0.30	0.56
	Mean		0.68	0.55	0.63	0.51	0.36	0.41
	Sdev		0.06	0.03	0.08	0.04	0.04	0.12
ParaSurf	1	7	0.78	0.53	0.56	0.54	0.38	0.51
	2	7	0.78	0.52	0.66	0.56	0.41	0.46
	3	8	0.82	0.52	0.59	0.57	0.42	0.39
	4	7	0.78	0.52	0.64	0.54	0.41	0.45
	5	7	0.80	0.53	0.57	0.55	0.39	0.48
	Mean		0.79	0.53	0.61	0.55	0.40	0.46
	Sdev		0.02	0.01	0.04	0.01	0.01	0.04

MUE_train_ and MUE_test_ = mean unsigned error on training and test sets; R^2^_train_ and R^2^_test_ = R^2^ on training and test sets; R^2^_cv_ = leave-one-out cross-validated R^2^.

**Table 4:** Mean predictions of committee of 10 two-hidden unit ANNs with highest evidence.

Descriptors	Training set	MUE_train_	MUE_test_	R^2^_train_	R^2^_test_
MOE	1	0.42	0.54	0.71	0.55
	2	0.42	0.62	0.72	0.47
	3	0.47	0.54	0.65	0.58
	4	0.45	0.53	0.65	0.65
	5	0.43	0.45	0.71	0.68
	Mean	0.44	0.54	0.69	0.58
	Sdev	0.02	0.06	0.03	0.09
Vsurf	1	0.43	0.62	0.71	0.47
	2	0.40	0.67	0.73	0.36
	3	0.41	0.64	0.73	0.43
	4	0.42	0.63	0.69	0.49
	5	0.44	0.54	0.69	0.57
	Mean	0.42	0.62	0.71	0.46
	Sdev	0.02	0.05	0.02	0.08
ParaSurf	1	0.39	0.53	0.75	0.58
	2	0.38	0.63	0.76	0.46
	3	0.40	0.56	0.74	0.45
	4	0.38	0.58	0.73	0.56
	5	0.40	0.52	0.75	0.59
	Mean	0.39	0.56	0.75	0.53
	Sdev	0.01	0.04	0.01	0.07

MUE_train_ and MUE_test_ = mean unsigned error on training and test sets; R^2^_train_ and R^2^_test_ = R^2^ on training and test sets.

**Table 5:** Ten most relevant descriptors in ANNs identified by ARD, ordered by mean rank over the 5 test sets. See Tables S4 and S5 for definition of MOE and Vsurf descriptors. ParaSurf descriptors are: polarizability (*molecular electronic polarizability*), FNmin (*minimum of electrostatic field normal to surface*), var.*balance (*product of total variance in molecular electrostatic potential and the balance parameter*), HARDrange (*range of local electron hardness*), MEPskew (*skewness of the molecular electrostatic potential*), MEPvar + (*variance of the positive molecular electrostatic potential*), meanMEP- (*mean of the negative molecular electrostatic potential*)), POLkurt (*kurtosis of the local polarizability*), EALkurt (*kurtosis of the local electron affinity*), FNmax (*maximum of electrostatic field normal to surface*).

MOE	Vsurf	ParaSurf
a_base	vsurf_R	polarizability
vsa_hyd	vsurf_G	FNmin
PC +	vsurf_EWmin1	var*balance
a_don	vsurf_CW2	HARDrange
rgyr	vsurf_CW1	MEPskew
vsa_pol	vsurf_HB4	MEPvar +
log *K*_*ow*_	vsurf_ID7	meanMEP −
rings	vsurf_IW5	POLkurt
a_acc	vsurf_HL2	EALkurt
Weight	vsurf_ID8	FNmax

## Appendix A Supplementary data

Supplementary material Image 2
