# Supplementary material for: Quantitative structure-property relationships for predicting sorption of pharmaceuticals to sewage sludge during waste water treatment processes
Source: Sci Total Environ. 2017 Feb 1;579:1512–20. doi: 10.1016/j.scitotenv.2016.11.156 (PMC5206221; doi:10.1016/j.scitotenv.2016.11.156)
Supplement: Supplementary file 1 — Supplementary material [file mmc1.doc]

**Supplementary Material**

**Quantitative structure-property relationships for predicting sorption of pharmaceuticals to sewage sludge during waste water treatment processes**

L. Berthod^ab^, D. C. Whitley*^b^, G. Roberts^a†^, A. Sharpe^a††^, R. Greenwood^c^ and G. A. Mills^b^

^a^AstraZeneca Global Environment, Alderley Park, Macclesfield, SK10 8TG, UK.

^b^School of Pharmacy and Biomedical Sciences, University of Portsmouth, St Michael’s Building, White Swan Road, Portsmouth, Hampshire, PO1 2DT, UK.

^c^School of Biological Sciences, University of Portsmouth, King Henry Building, King Henry I Street, Portsmouth, Hampshire, PO1 2DY, UK.

Current addresses:

†Regulatory Science Associates at Kip Marina, Inverkip, Renfrewshire, PA16 0AS, UK.

††AstraZeneca Discovery Sciences, Darwin Building, Block 310, Cambridge Science Park, Milton Road, Cambridge, CB4 0FZ.

*Corresponding author. E-mail: david.whitley@port.ac.uk, Tel: +44 (0)2392 845 080

**1. Methods**

**1.1 Experimental log *K_d_* values**

Table S1 lists the dataset of 297 experimental log *K_d_* values for 148 APIs and major metabolites found from a review of the literature, together with internal AstraZeneca results. Experimental pH was reported for 142 (48%) of the measurements, ranging from 6.30-7.74, with mean 7.03. The analytical method used for the experimental measurements was liquid chromatography/mass spectrometry, apart from Halling-Sørensen (2000), Clara et al. (2004) and Berthod (2015) who used liquid chromatography with ultra violet detection, and Urase et al. (2005) and Stasinakis et al. (2013) who used gas chromatography/mass spectrometry.

**Table S1.** Experimental log *K_d_* values with sludge type and experimental pH, where specified.

| **Compound** | **ID** | **Reference** | **Sludge type** | **log *K_d_*** | **pH** |
| --- | --- | --- | --- | --- | --- |
| 10,11-Dihydro-10,11-dihydroxy-carbamazepine | 1 | Wick et al. (2009) | activated | 0.85 |  |
| 10,11-Dihydrocarbamazepine | 2 | Wick et al. (2009) | activated | 1.08 |  |
| Acetaminophen | 3 | Berthod (2015) | activated | 2.52 | 7.4 |
|  | 4 | Radjenović et al. (2009) | primary | 0.83 |  |
|  | 5 | Radjenović et al. (2009) | activated | 3.06 |  |
| Alfuzosin | 6 | Hörsing et al. (2011) | primary | 3.26 | 7.0 |
| Allopurinol | 7 | Berthod (2015) | activated | 2.70 | 7.4 |
| Amitriptyline | 8 | Hörsing et al. (2011) | primary | 3.61 | 7.0 |
|  | 9 | Hyland et al. (2012) | activated | 2.87 | 6.3 |
|  | 10 | Lajeunesse et al. (2012) | primary | 4.41 | 7.0 |
|  | 11 | Stevens-Garmon et al. (2011) | activated | 3.66 | 7.0 |
|  | 12 | Stevens-Garmon et al. (2011) | primary | 3.76 |  |
| Amlodipine | 13 | Berthod (2015) | activated | 3.36 | 7.4 |
|  | 14 | Yan et al. (2014) | primary | 2.20 |  |
|  | 15 | Yan et al. (2014) | primary | 2.02 |  |
| Amoxicillin | 16 | Berthod (2015) | activated | 3.26 | 7.4 |
| Androstenedione | 17 | Stevens-Garmon et al. (2011) | activated | 2.19 | 7.0 |
|  | 18 | Stevens-Garmon et al. (2011) | primary | 2.24 |  |
| Androsterone | 19 | Stevens-Garmon et al. (2011) | activated | 2.76 | 7.0 |
|  | 20 | Stevens-Garmon et al. (2011) | primary | 2.73 |  |
| Aspirin | 21 | Berthod (2015) | activated | 1.60 | 7.4 |
| Atenolol | 22 | Hörsing et al. (2011) | primary | 2.66 | 7.0 |
|  | 23 | Radjenović et al. (2009) | primary | 1.98 |  |
|  | 24 | Radjenović et al. (2009) | activated | 1.81 |  |
|  | 25 | Stevens-Garmon et al. (2011) | primary | 1.66 |  |
|  | 26 | Wick et al. (2009) | activated | 1.58 |  |
| Atorvastatin | 27 | Stevens-Garmon et al. (2011) | activated | 2.30 | 7.0 |

| Atorvastatin | 28 | Stevens-Garmon et al. (2011) | primary | 2.33 |  |
| --- | --- | --- | --- | --- | --- |
|  | 29 | Yan et al. (2014) | primary | 1.94 |  |
|  | 30 | Yan et al. (2014) | primary | 2.10 |  |
| Atracurium | 31 | Hörsing et al. (2011) | primary | 2.54 | 7.0 |
| Azelastine | 32 | Hörsing et al. (2011) | primary | 3.81 | 7.0 |
| Azithromycin | 33 | Göbel et al. (2005) | activated | 2.58 |  |
|  | 34 | Yan et al. (2014) | primary | 2.55 |  |
|  | 35 | Yan et al. (2014) | primary | 1.55 |  |
| Bendroflumethiazide | 36 | Berthod (2015) | activated | 2.88 | 7.4 |
| Bezafibrate | 37 | Yan et al. (2014) | primary | 1.11 |  |
|  | 38 | Yan et al. (2014) | primary | 1.36 |  |
| Bicalutamide | 39 | AstraZeneca | activated | 2.15 |  |
| Biperiden | 40 | Hörsing et al. (2011) | primary | 2.91 | 7.0 |
| Bisoprolol | 41 | Wick et al. (2009) | activated | 1.60 |  |
| Bupropion | 42 | Hörsing et al. (2011) | primary | 1.93 | 7.0 |
| Candesartan | 43 | AstraZeneca | activated | 1.23 |  |
| Carbamazepine | 44 | Berthod (2015) | activated | 1.89 | 7.4 |
|  | 45 | Hyland et al. (2012) | activated | 1.95 | 6.3 |
|  | 46 | Lajeunesse et al. (2012) | primary | 2.34 | 7.0 |
|  | 47 | Radjenović et al. (2009) | primary | 2.50 |  |
|  | 48 | Radjenović et al. (2009) | activated | 2.13 |  |
|  | 49 | Stevens-Garmon et al. (2011) | activated | 1.70 | 7.0 |
|  | 50 | Stevens-Garmon et al. (2011) | primary | 1.81 |  |
|  | 51 | Ternes et al. (2004) | activated | 0.08 | 7.5 |
|  | 52 | Urase et al. (2005) | activated | 1.82 | 6.7 |
|  | 53 | Wick et al. (2009) | activated | 1.23 |  |
|  | 54 | Yan et al. (2014) | primary | 1.66 |  |
|  | 55 | Yan et al. (2014) | primary | 1.61 |  |
| Cediranib | 56 | AstraZeneca | activated | 3.89 |  |
| Ceftadizime | 57 | Berthod (2015) | activated | 2.79 | 7.4 |
| Celiprolol | 58 | Wick et al. (2009) | activated | 1.93 |  |
| Chlorprothixene | 59 | Hörsing et al. (2011) | primary | 4.58 | 7.0 |
| Cimetidine | 60 | Hyland et al. (2012) | activated | 2.51 | 6.3 |
| Ciprofloxacin | 61 | Halling-Sørensen (2000) | activated | 2.62 |  |
|  | 62 | Jia et al. (2012) | activated | 4.51 | 7.7 |
| Citalopram | 63 | Hörsing et al. (2011) | primary | 2.73 | 7.0 |
|  | 64 | Lajeunesse et al. (2012) | primary | 4.40 | 7.0 |
| Clarithromycin | 65 | Göbel et al. (2005) | activated | 2.42 |  |
| Clofibric acid | 66 | AstraZeneca | activated | 1.34 |  |
|  | 67 | Berthod (2015) | activated | 1.30 | 7.4 |
|  | 68 | Ternes et al. (2004) | activated | 0.68 | 7.5 |
|  | 69 | Urase et al. (2005) | activated | 1.46 |  |
| Clofibric acid | 70 | Yan et al. (2014) | primary | 1.53 |  |
|  | 71 | Yan et al. (2014) | primary | 1.57 |  |
| Clomipramine | 72 | Berthod (2015) | activated | 3.11 | 7.4 |
|  | 73 | Hörsing et al. (2011) | primary | 4.23 | 7.0 |
| Clonazepam | 74 | Hörsing et al. (2011) | primary | 2.76 | 7.0 |
| Clotrimazole | 75 | Berthod (2015) | activated | 3.54 | 7.4 |
|  | 76 | Hörsing et al. (2011) | primary | 4.51 | 7.0 |
| Clozapine | 77 | Stevens-Garmon et al. (2011) | activated | 3.22 | 7.0 |
|  | 78 | Stevens-Garmon et al. (2011) | primary | 3.24 |  |
| Codeine | 79 | Wick et al. (2009) | activated | 1.15 |  |
| Cyclophosphamide | 80 | Ternes et al. (2004) | primary | 1.74 | 6.6 |
|  | 81 | Ternes et al. (2004) | activated | 0.38 | 7.5 |
| Cyproheptadine | 82 | Hörsing et al. (2011) | primary | 4.04 | 7.0 |
| Dapagliflozin | 83 | AstraZeneca | activated | 1.71 |  |
| Desloratadine | 84 | Hörsing et al. (2011) | primary | 3.57 | 7.0 |
| Desmethylmirtazepine | 85 | Lajeunesse et al. (2012) | primary | 3.73 | 7.0 |
| Desmethylsertraline | 86 | Lajeunesse et al. (2012) | primary | 4.49 | 7.0 |
| Desmethylvenlafaxine | 87 | Lajeunesse et al. (2012) | primary | 2.15 | 7.0 |
| Diazapam | 88 | Hyland et al. (2012) | activated | 2.14 | 6.3 |
|  | 89 | Stevens-Garmon et al. (2011) | activated | 2.38 | 7.0 |
| Diazepam | 90 | Stevens-Garmon et al. (2011) | primary | 2.46 |  |
|  | 91 | Ternes et al. (2004) | primary | 1.64 | 6.6 |
|  | 92 | Ternes et al. (2004) | activated | 1.32 | 7.5 |
|  | 93 | Wick et al. (2009) | activated | 1.72 |  |
| Diclofenac | 94 | AstraZeneca | activated | 2.11 |  |
|  | 95 | Berthod (2015) | activated | 1.96 | 7.4 |
|  | 96 | Hyland et al. (2012) | activated | 2.18 | 6.3 |
|  | 97 | Radjenović et al. (2009) | primary | 2.29 |  |
|  | 98 | Radjenović et al. (2009) | activated | 2.07 |  |
| Diclofenac | 99 | Stasinakis et al. (2013) | primary | 2.66 |  |
|  | 100 | Ternes et al. (2004) | primary | 2.66 | 6.6 |
|  | 101 | Ternes et al. (2004) | activated | 1.20 | 7.5 |
|  | 102 | Urase et al. (2005) | activated | 1.51 | 6.7 |
|  | 103 | Yan et al. (2014) | primary | 2.03 |  |
|  | 104 | Yan et al. (2014) | primary | 2.02 |  |
| Dicycloverine | 105 | Hörsing et al. (2011) | primary | 3.15 | 7.0 |
| Dihydrocodeine | 106 | Wick et al. (2009) | activated | 1.08 |  |
| Dilantin | 107 | Hyland et al. (2012) | activated | 2.49 | 6.3 |
|  | 108 | Stevens-Garmon et al. (2011) | activated | 1.91 | 7.0 |
|  | 109 | Stevens-Garmon et al. (2011) | primary | 1.65 |  |
| Diphenhydramine | 110 | Hyland et al. (2012) | activated | 2.50 | 6.3 |
| Donepezil | 111 | Hörsing et al. (2011) | primary | 3.56 | 7.0 |
| Doxepin | 112 | Wick et al. (2009) | activated | 2.14 |  |
| Duloxetine | 113 | Hörsing et al. (2011) | primary | 4.11 | 7.0 |
| Enrofloxacin | 114 | Jia et al. (2012) | activated | 4.55 | 7.7 |
| Ergocalciferol | 115 | Berthod (2015) | activated | 4.28 | 7.4 |
| Erythromycin | 116 | Radjenović et al. (2009) | primary | 2.49 |  |
|  | 117 | Radjenović et al. (2009) | activated | 1.87 |  |
|  | 118 | Yan et al. (2014) | primary | 1.57 |  |
|  | 119 | Yan et al. (2014) | primary | 1.42 |  |
| Esomeprazole | 120 | AstraZeneca | activated | 1.68 |  |
| Estradiol | 121 | Andersen et al. (2004) | activated | 2.68 |  |
|  | 122 | Clara et al. (2004) | activated | 2.84 |  |
|  | 123 | Stevens-Garmon et al. (2011) | activated | 2.89 | 7.0 |
|  | 124 | Stevens-Garmon et al. (2011) | primary | 2.75 |  |
|  | 125 | Urase et al. (2005) | activated | 3.18 | 6.7 |
| Estriol | 126 | Stevens-Garmon et al. (2011) | activated | 1.80 | 7.0 |
|  | 127 | Stevens-Garmon et al. (2011) | primary | 1.76 |  |
| Estrone | 128 | Andersen et al. (2004) | activated | 2.60 |  |
| Estrone | 129 | Stevens-Garmon et al. (2011) | activated | 2.81 | 7.0 |
|  | 130 | Stevens-Garmon et al. (2011) | primary | 2.80 |  |
|  | 131 | Urase et al. (2005) | activated | 2.31 | 6.7 |
| Ethinylestradiol | 132 | Andersen et al. (2004) | activated | 2.77 |  |
|  | 133 | Clara et al. (2004) | activated | 2.84 |  |
|  | 134 | Stevens-Garmon et al. (2011) | activated | 3.19 | 7.0 |
|  | 135 | Stevens-Garmon et al. (2011) | primary | 3.01 |  |
|  | 136 | Ternes et al. (2004) | primary | 2.44 | 6.6 |
|  | 137 | Ternes et al. (2004) | activated | 2.54 | 7.5 |
|  | 138 | Urase et al. (2005) | activated | 2.67 | 6.7 |
| Ezetimibe | 139 | Hörsing et al. (2011) | primary | 3.36 | 7.0 |
| Felodipine | 140 | AstraZeneca | activated | 3.45 |  |
| Fenoprofen | 141 | Urase et al. (2005) | activated | 1.76 | 6.7 |
| Fexofenadine | 142 | Hörsing et al. (2011) | primary | 3.43 | 7.0 |
| Fleroxacin | 143 | Jia et al. (2012) | activated | 4.18 | 7.7 |
| Fluoxetine | 144 | Hörsing et al. (2011) | primary | 4.00 | 7.0 |
|  | 145 | Hyland et al. (2012) | activated | 3.08 | 6.3 |
|  | 146 | Lajeunesse et al. (2012) | primary | 4.64 | 7.0 |
| Flutamide | 147 | Berthod (2015) | activated | 2.71 | 7.4 |
|  | 148 | Hörsing et al. (2011) | primary | 3.18 | 7.0 |
| Gatifloxacin | 149 | Jia et al. (2012) | activated | 4.17 | 7.7 |
| Gefitinib | 150 | AstraZeneca | activated | 3.27 |  |
| Gemfibrozil | 151 | Hyland et al. (2012) | activated | 2.11 | 6.3 |
|  | 152 | Radjenović et al. (2009) | primary | 1.36 |  |
|  | 153 | Radjenović et al. (2009) | activated | 1.29 |  |
|  | 154 | Stevens-Garmon et al. (2011) | activated | 1.65 | 7.0 |
|  | 155 | Stevens-Garmon et al. (2011) | primary | 1.65 |  |
|  | 156 | Urase et al. (2005) | activated | 2.00 | 6.7 |
| Glibenclamide | 157 | Hörsing et al. (2011) | primary | 3.56 | 7.0 |
| Glibenclamide | 158 | Radjenović et al. (2009) | primary | 2.45 |  |
|  | 159 | Radjenović et al. (2009) | activated | 2.38 |  |
| Glimepiride | 160 | Hörsing et al. (2011) | primary | 3.32 | 7.0 |
| Haloperidol | 161 | Hörsing et al. (2011) | primary | 4.00 | 7.0 |
| Hydrochlorothiazide | 162 | Berthod (2015) | activated | 1.30 | 7.4 |
|  | 163 | Radjenović et al. (2009) | primary | 1.41 |  |
|  | 164 | Radjenović et al. (2009) | activated | 1.31 |  |
| Hydrocodone | 165 | Hyland et al. (2012) | activated | 2.03 | 6.3 |
| Hydroxyzine | 166 | Hörsing et al. (2011) | primary | 3.08 | 7.0 |
|  | 167 | Stevens-Garmon et al. (2011) | activated | 2.91 | 7.0 |
|  | 168 | Stevens-Garmon et al. (2011) | primary | 2.89 |  |
| Ibuprofen | 169 | AstraZeneca | activated | 0.93 |  |
|  | 170 | Hyland et al. (2012) | activated | 2.32 | 6.3 |
|  | 171 | Radjenović et al. (2009) | primary | 0.98 |  |
|  | 172 | Ternes et al. (2004) | activated | 0.85 | 7.5 |
|  | 173 | Urase et al. (2005) | activated | 1.90 | 6.7 |
|  | 174 | Yan et al. (2014) | primary | 1.16 |  |
|  | 175 | Yan et al. (2014) | primary | 0.92 |  |
| Ifosfamide | 176 | Ternes et al. (2004) | primary | 1.34 | 6.6 |
|  | 177 | Ternes et al. (2004) | activated | 0.15 | 7.5 |
| Indomethacin | 178 | Urase et al. (2005) | activated | 1.59 | 6.7 |
| Irbesartan | 179 | Hörsing et al. (2011) | primary | 2.85 | 7.0 |
| Ketoconazole | 180 | Hörsing et al. (2011) | primary | 3.99 | 7.0 |
| Ketoprofen | 181 | Hyland et al. (2012) | activated | 2.25 | 6.3 |
|  | 182 | Radjenović et al. (2009) | primary | 2.35 |  |
|  | 183 | Radjenović et al. (2009) | activated | 1.20 |  |
|  | 184 | Urase et al. (2005) | activated | 1.46 | 6.7 |
| Lansoprazole | 185 | Berthod (2015) | activated | 2.16 | 7.4 |
| Lesinurad | 186 | Berthod (2015) | activated | 1.59 | 7.4 |
| Levothyroxine | 187 | Berthod (2015) | activated | 3.86 | 7.4 |
| Lisinopril | 188 | Berthod (2015) | activated | 3.64 | 7.4 |
| Lomefloxacin | 189 | Jia et al. (2012) | activated | 4.16 | 7.7 |
| Loperamide | 190 | Hörsing et al. (2011) | primary | 4.15 | 7.0 |
| Loratadine | 191 | Radjenović et al. (2009) | primary | 3.37 |  |
|  | 192 | Radjenović et al. (2009) | activated | 3.52 |  |
| Maprotiline | 193 | Hörsing et al. (2011) | primary | 3.83 | 7.0 |
| Mecillinam | 194 | Berthod (2015) | activated | 1.52 | 7.4 |
|  | 195 | Halling-Sørensen (2000) | activated | 1.74 |  |
| Mefenamic acid | 196 | Radjenović et al. (2009) | primary | 2.47 |  |
|  | 197 | Radjenović et al. (2009) | activated | 2.64 |  |
| Meprobamate | 198 | Stevens-Garmon et al. (2011) | primary | 1.62 |  |
| Metformin | 199 | Berthod (2015) | activated | 1.47 | 7.4 |
| Methadone | 200 | Wick et al. (2009) | activated | 1.88 |  |
| Metoprolol | 201 | Wick et al. (2009) | activated | 1.81 |  |
|  | 202 | Yan et al. (2014) | primary | 1.50 |  |
|  | 203 | Yan et al. (2014) | primary | 1.75 |  |
| Mianserin | 204 | Hörsing et al. (2011) | primary | 3.48 | 7.0 |
| Mirtazapine | 205 | Lajeunesse et al. (2012) | primary | 3.52 | 7.0 |
| Morphine | 206 | Wick et al. (2009) | activated | 1.08 |  |
| Moxifloxacin | 207 | Jia et al. (2012) | activated | 4.18 | 7.7 |
|  | 208 | Yan et al. (2014) | primary | 2.73 |  |
|  | 209 | Yan et al. (2014) | primary | 2.56 |  |

| Naproxen | 210 | Hyland et al. (2012) | activated | 2.16 | 6.3 |
| --- | --- | --- | --- | --- | --- |
|  | 211 | Urase et al. (2005) | activated | 1.38 | 6.7 |
| Nefazodone | 212 | Hörsing et al. (2011) | primary | 4.15 | 7.0 |
| Nordiazapam | 213 | Wick et al. (2009) | activated | 1.81 |  |
| Norfloxacin | 214 | Jia et al. (2012) | activated | 4.52 | 7.7 |
|  | 215 | Yan et al. (2014) | primary | 2.67 |  |
|  | 216 | Yan et al. (2014) | primary | 2.05 |  |
| Norfluoxetine | 217 | Lajeunesse et al. (2012) | primary | 4.20 | 7.0 |
| Nortriptyline | 218 | Lajeunesse et al. (2012) | primary | 4.46 | 7.0 |
| Ofloxacin | 219 | Jia et al. (2012) | activated | 4.25 | 7.7 |
|  | 220 | Yan et al. (2014) | primary | 2.67 |  |
| Ofloxacin | 221 | Yan et al. (2014) | primary | 2.14 |  |
| Olaparib | 222 | AstraZeneca | activated | 1.40 |  |
| Omeprazole | 223 | Stevens-Garmon et al. (2011) | activated | 2.03 | 7.0 |
|  | 224 | Stevens-Garmon et al. (2011) | primary | 2.11 |  |
| Oxazepam | 225 | Hörsing et al. (2011) | primary | 3.90 | 7.0 |
|  | 226 | Wick et al. (2009) | activated | 1.11 |  |
| Oxycodone | 227 | Wick et al. (2009) | activated | 1.15 |  |
| Oxytetracycline | 228 | AstraZeneca | activated | 3.85 |  |
|  | 229 | Berthod (2015) | activated | 3.67 | 7.4 |
| Paroxetine | 230 | Hörsing et al. (2011) | primary | 4.15 | 7.0 |
|  | 231 | Lajeunesse et al. (2012) | primary | 4.36 | 7.0 |
| Pipemidic acid | 232 | Jia et al. (2012) | activated | 4.09 | 7.7 |
| Pizotifen | 233 | Hörsing et al. (2011) | primary | 3.67 | 7.0 |
| Primidone | 234 | Stevens-Garmon et al. (2011) | primary | 1.65 |  |
|  | 235 | Wick et al. (2009) | activated | 0.85 |  |
| Progesterone | 236 | Hörsing et al. (2011) | primary | 2.88 | 7.0 |
| Propranolol | 237 | AstraZeneca | activated | 2.62 |  |
|  | 238 | Radjenović et al. (2009) | primary | 2.81 |  |
|  | 239 | Radjenović et al. (2009) | activated | 2.56 |  |
|  | 240 | Wick et al. (2009) | activated | 2.54 |  |
| Propyphenazone | 241 | Urase et al. (2005) | activated | 1.18 | 6.7 |
| Quetiapine | 242 | AstraZeneca | activated | 2.53 |  |
| Ramipril | 243 | Berthod (2015) | activated | 1.56 | 7.4 |
| Repaglinide | 244 | Hörsing et al. (2011) | primary | 2.23 | 7.0 |
| Risperidone | 245 | Hörsing et al. (2011) | primary | 3.28 | 7.0 |
|  | 246 | Stevens-Garmon et al. (2011) | activated | 2.94 | 7.0 |
|  | 247 | Stevens-Garmon et al. (2011) | primary | 2.98 |  |
| Roxithromycin | 248 | Berthod (2015) | activated | 2.54 | 7.4 |
|  | 249 | Yan et al. (2014) | primary | 1.88 |  |
|  | 250 | Yan et al. (2014) | primary | 1.57 |  |
| Salbutamol | 251 | Berthod (2015) | activated | 2.19 | 7.4 |
| Saxagliptin | 252 | AstraZeneca | activated | 1.50 |  |
| Selumetinib | 253 | Berthod (2015) | activated | 2.10 | 7.4 |
| Sertraline | 254 | Lajeunesse et al. (2012) | primary | 4.92 | 7.0 |
|  | 255 | Hörsing et al. (2011) | primary | 4.54 | 7.0 |
| Simvastatin | 256 | Berthod (2015) | activated | 3.71 | 7.4 |
|  | 257 | Yan et al. (2014) | primary | 2.85 |  |
|  | 258 | Yan et al. (2014) | primary | 3.08 |  |
| Sotalol | 259 | Wick et al. (2009) | activated | 1.26 |  |
| Sparfloxacin | 260 | Jia et al. (2012) | activated | 4.58 | 7.7 |
| Sulfadiazine | 261 | Yan et al. (2014) | primary | 1.13 |  |
|  | 262 | Yan et al. (2014) | primary | 0.66 |  |
| Sulfamethazine | 263 | Yan et al. (2014) | primary | 1.24 |  |
|  | 264 | Yan et al. (2014) | primary | 1.12 |  |
| Sulfamethoxazole | 265 | Göbel et al. (2005) | activated | 2.41 |  |
|  | 266 | Hörsing et al. (2011) | primary | 2.51 | 7.0 |
|  | 267 | Hyland et al. (2012) | activated | 2.43 | 6.3 |
|  | 268 | Radjenović et al. (2009) | primary | 0.51 |  |
|  | 269 | Radjenović et al. (2009) | activated | 1.89 |  |
|  | 270 | Yan et al. (2014) | primary | 0.63 |  |
|  | 271 | Yan et al. (2014) | primary | 1.30 |  |
| Sulfapyridine | 272 | Göbel et al. (2005) | activated | 2.47 |  |
| Telmisartan | 273 | Hörsing et al. (2011) | primary | 3.11 | 7.0 |
| Testosterone | 274 | Stevens-Garmon et al. (2011) | activated | 2.20 | 7.0 |
|  | 275 | Stevens-Garmon et al. (2011) | primary | 2.25 |  |
| Ticagrelor | 276 | AstraZeneca | activated | 3.20 |  |
| Tramadol | 277 | Hörsing et al. (2011) | primary | 2.04 | 7.0 |
|  | 278 | Wick et al. (2009) | activated | 1.67 |  |
| Triclocarban | 279 | Hyland et al. (2012) | activated | 4.41 | 6.3 |
| Triclosan | 280 | Hyland et al. (2012) | activated | 3.59 | 6.3 |
| Trimethoprim | 281 | Göbel et al. (2005) | activated | 2.32 |  |
|  | 282 | Halling-Sørensen (2000) | activated | 1.88 |  |
|  | 283 | Hörsing et al. (2011) | primary | 2.59 | 7.0 |
|  | 284 | Hyland et al. (2012) | activated | 2.30 | 6.3 |
|  | 285 | Radjenović et al. (2009) | primary | 2.63 |  |
| Trimethoprim | 286 | Radjenović et al. (2009) | activated | 2.40 |  |
|  | 287 | Stevens-Garmon et al. (2011) | activated | 2.08 | 7.0 |
|  | 288 | Stevens-Garmon et al. (2011) | primary | 2.40 |  |
|  | 289 | Yan et al. (2014) | primary | 2.12 |  |
|  | 290 | Yan et al. (2014) | primary | 1.93 |  |
| Vandetanib | 291 | AstraZeneca | activated | 3.92 |  |
| Venlafaxine | 292 | Lajeunesse et al. (2012) | primary | 3.18 | 7.0 |
| Verapamil | 293 | Hörsing et al. (2011) | primary | 3.26 | 7.0 |
|  | 294 | Stevens-Garmon et al. (2011) | activated | 3.18 | 7.0 |
|  | 295 | Stevens-Garmon et al. (2011) | primary | 3.22 |  |
| Zibotentan | 296 | AstraZeneca | activated | -0.70 |  |
|  | 297 | Berthod (2015) | activated | 1.18 | 7.4 |

**1.2 Chemical structures**

Structures in structure data format (SDF) for the 148 APIs at the pH at which the log *K_d_* values were measured, where reported, otherwise at pH 7.4, were obtained from the ChemAxon website (http://www.chemicalize.org/). Their canonical SMILES (Weininger, 1988; Weininger et al., 1989) generated by MOE (MOE, 2012) are shown in Table S2.

**Table S2.** Canonical SMILES generated by MOE for dominant species at experimental pH, where reported, otherwise at pH 7.4.

| **Compound** | **SMILES** |
| --- | --- |
| 10,11-Dihydro-10,11-dihydroxy-carbamazepine | OC1c2c(N(c3c(cccc3)C1O)C(=O)N)cccc2 |
| 10,11-Dihydro-carbamazepine | O=C(N)N1c2c(CCc3c1cccc3)cccc2 |
| Acetaminophen | Oc1ccc(NC(=O)C)cc1 |
| Alfuzosin | O1CCCC1C(=O)NCCCN(C)c1[nH+]c2c(cc(OC)c(OC)c2)c(n1)N |
| Allopurinol | Oc1[nH]cnc-2nncc1-2 |
| Amitriptyline | [NH+](CC\C=C/1\c2c(CCc3c\1cccc3)cccc2)(C)C |
| Amlodipine | Clc1ccccc1C1C(C(OCC)=O)=C(NC(C)=C1C(OC)=O)COCC[NH3+] |
| Amoxicillin | S1[C@H]2N([C@@H](C(=O)[O-])C1(C)C)C(=O)[C@H]2NC(=O)[C@H]([NH3+])c1ccc(O)cc1 |
| Androstenedione | O=C1CC[C@H]2[C@H]3[C@H](CC[C@]12C)[C@@]1(C(=CC(=O)CC1)CC3)C |
| Androsterone | O=C1CC[C@H]2[C@H]3[C@H](CC[C@]12C)[C@@]1([C@H](C[C@H](O)CC1)CC3)C |
| Aspirin | O(C(=O)C)c1ccccc1C(=O)[O-] |
| Atenolol | O(CC(O)C[NH2+]C(C)C)c1ccc(cc1)CC(=O)N |
| Atorvastatin | Fc1ccc(cc1)-c1n(CC[C@@H](O)C[C@@H](O)CC(=O)[O-])c(C(C)C)c(C(=O)Nc2ccccc2)c1-c1ccccc1 |
| Atracurium | O(C)c1cc(ccc1OC)CC1[N+](CCc2c1cc(OC)c(OC)c2)(CCC(OCCCCCOC(=O)CC[N+]1(CCc2c(cc(OC)c(OC)c2)C1Cc1cc(OC)c(OC)cc1)C)=O)C |
| Azelastine | Clc1ccc(cc1)CC1=NN(C2CCC[NH+](CC2)C)C(=O)c2c1cccc2 |
| Azithromycin | O1[C@H](CC)[C@](O)(C)[C@H](O)[C@H]([NH+](C[C@@H](C[C@](O)(C)[C@H](O[C@@H]2O[C@@H](C[C@H]([NH+](C)C)[C@H]2O)C)[C@@H](C)[C@H](O[C@@H]2O[C@@H](C)[C@H](O)[C@](OC)(C2)C)[C@@H](C)C1=O)C)C)C |
| Bendroflumethiazide | S(=O)(=O)(N)c1cc2S(=O)(=O)NC(Nc2cc1C(F)(F)F)Cc1ccccc1 |
| Bezafibrate | Clc1ccc(cc1)C(=O)NCCc1ccc(OC(C(=O)[O-])(C)C)cc1 |
| Bicalutamide | S(=O)(=O)(CC(O)(C(=O)Nc1cc(C(F)(F)F)c(cc1)C#N)C)c1ccc(F)cc1 |
| Biperiden | OC(CC[NH+]1CCCCC1)(C1C2CC(C1)C=C2)c1ccccc1 |
| Bisoprolol | O(CC(O)C[NH2+]C(C)C)c1ccc(cc1)COCCOC(C)C |
| Bupropion | Clc1cc(ccc1)C(=O)C([NH2+]C(C)(C)C)C |
| Candesartan | O(CC)c1nc2c(n1Cc1ccc(cc1)-c1ccccc1-c1n[n-]nn1)c(ccc2)C(=O)[O-] |
| Carbamazepine | O=C(N)N1c2c(C=Cc3c1cccc3)cccc2 |
| Cediranib | Fc1c2cc([nH]c2ccc1Oc1ncnc2c1cc(OC)c(OCCC[NH+]1CCCC1)c2)C |
| Ceftazidime | s1cc(nc1N)/C(=N/OC(C(=O)[O-])(C)C)/C(=O)N[C@H]1[C@H]2SCC(C[n+]3ccccc3)=C(N2C1=O)C(=O)[O-] |
| Celiprolol | O(CC(O)C[NH2+]C(C)(C)C)c1ccc(NC(=O)N(CC)CC)cc1C(=O)C |
| Chlorprothixene | Clc1cc\2c(Sc3c(cccc3)/C/2=C/CC[NH+](C)C)cc1 |
| Cimetidine | S(Cc1[nH]c[nH+]c1C)CCN\C(=N\C)\NC#N |
| Ciprofloxacin | Fc1cc2c(N(C=C(C(=O)[O-])C2=O)C2CC2)cc1N1CC[NH2+]CC1 |
| Citalopram | Fc1ccc(cc1)C1(OCc2c1ccc(c2)C#N)CCC[NH+](C)C |
| Clarithromycin | O1[C@H](CC)[C@](O)(C)[C@H](O)[C@@H](C)C(=O)[C@@H](C[C@](OC)(C)[C@H](O[C@@H]2O[C@@H](C[C@H]([NH+](C)C)[C@H]2O)C)[C@@H](C)[C@H](O[C@@H]2O[C@@H](C)[C@H](O)[C@](OC)(C2)C)[C@@H](C)C1=O)C |
| Clofibric_acid | Clc1ccc(OC(C(=O)[O-])(C)C)cc1 |
| Clomipramine | Clc1cc2N(c3c(CCc2cc1)cccc3)CCC[NH+](C)C |
| Clonazepam | Clc1ccccc1C1=NCC(=O)Nc2c1cc([N+](=O)[O-])cc2 |
| Clotrimazole | Clc1ccccc1C(n1ccnc1)(c1ccccc1)c1ccccc1 |
| Clozapine | Clc1cc2N=C(N3CC[NH+](CC3)C)c3c(Nc2cc1)cccc3 |
| Codeine | O1[C@@H]2[C@]34[C@H]([C@H]([NH+](CC3)C)Cc3c4c1c(OC)cc3)C=C[C@@H]2O |
| Cyclophosphamide | ClCCN(P1(OCCCN1)=O)CCCl |
| Cyproheptadine | [NH+]1(CCC(CC1)=C1c2c(C=Cc3c1cccc3)cccc2)C |
| Dapagliflozin | Clc1ccc(cc1Cc1ccc(OCC)cc1)[C@@H]1O[C@H](CO)[C@@H](O)[C@H](O)[C@H]1O |
| Desloratadine | Clc1cc2c(cc1)\C(\c1ncccc1CC2)=C\1/CC[NH2+]CC/1 |
| Desmethylmirtazapine | [NH2+]1CC2N(CC1)c1ncccc1Cc1c2cccc1 |
| Desmethylsertraline | Clc1cc(ccc1Cl)[C@@H]1CC[C@H]([NH3+])c2c1cccc2 |
| Desvenlafaxine | OC1(CCCCC1)C(C[NH+](C)C)c1ccc(O)cc1 |
| Diazepam | Clc1cc2c(N(C)C(=O)CN=C2c2ccccc2)cc1 |
| Diclofenac | Clc1cccc(Cl)c1Nc1ccccc1CC(=O)[O-] |
| Dicycloverine | O(C(=O)C1(CCCCC1)C1CCCCC1)CC[NH+](CC)CC |
| Dihydrocodeine | O1[C@@H]2[C@]34[C@H]([C@H]([NH+](CC3)C)Cc3c4c1c(OC)cc3)CC[C@@H]2O |
| Dilantin | O=C1NC(=O)NC1(c1ccccc1)c1ccccc1 |
| Diphenhydramine | O(C(c1ccccc1)c1ccccc1)CC[NH+](C)C |
| Donepezil | O(C)c1cc2c(CC(CC3CC[NH+](CC3)Cc3ccccc3)C2=O)cc1OC |
| Doxepin | O1Cc2c(cccc2)\C(\c2c1cccc2)=C\CC[NH+](C)C |
| Duloxetine | s1cccc1[C@@H](Oc1c2c(ccc1)cccc2)CC[NH2+]C |
| Enrofloxacin | Fc1cc2c(N(C=C(C(=O)[O-])C2=O)C2CC2)cc1N1CCN(CC1)CC |
| Ergocalciferol | O[C@@H]1C\C(=C\C=C/2\[C@@H]3CC[C@H]([C@@H](\C=C\[C@@H](C(C)C)C)C)[C@]3(CCC\2)C)\C(CC1)=C |
| Erythromycin | O1[C@H](CC)[C@](O)(C)[C@H](O)[C@@H](C)C(=O)[C@@H](C[C@](O)(C)[C@H](O[C@@H]2O[C@@H](C[C@H]([NH+](C)C)[C@H]2O)C)[C@@H](C)[C@H](O[C@@H]2O[C@@H](C)[C@H](O)[C@](OC)(C2)C)[C@@H](C)C1=O)C |
| Esomeprazole | S(=O)(Cc1ncc(C)c(OC)c1C)c1[nH]c2c(n1)cc(OC)cc2 |
| Estradiol | Oc1cc2CC[C@H]3[C@@H]4CC[C@H](O)[C@]4(CC[C@@H]3c2cc1)C |
| Estriol | Oc1cc2CC[C@H]3[C@@H]4C[C@@H](O)[C@H](O)[C@]4(CC[C@@H]3c2cc1)C |
| Estrone | Oc1cc2CC[C@H]3[C@@H]4CCC(=O)[C@]4(CC[C@@H]3c2cc1)C |
| Ethinylestradiol | Oc1cc2CC[C@H]3[C@@H]4CC[C@@](O)(C#C)[C@]4(CC[C@@H]3c2cc1)C |
| Ezetimibe | Fc1ccc(cc1)[C@@H](O)CC[C@@H]1[C@H](N(C1=O)c1ccc(F)cc1)c1ccc(O)cc1 |
| Felodipine | Clc1c(cccc1Cl)C1C(C(OCC)=O)=C(NC(C)=C1C(OC)=O)C |
| Fenoprofen | O(c1cc(ccc1)C(C(=O)[O-])C)c1ccccc1 |
| Fexofenadine | OC(C1CC[NH+](CC1)CCCC(O)c1ccc(cc1)C(C(=O)[O-])(C)C)(c1ccccc1)c1ccccc1 |
| Fleroxacin | Fc1c2N(C=C(C(=O)[O-])C(=O)c2cc(F)c1N1CCN(CC1)C)CCF |
| Fluoxetine | FC(F)(F)c1ccc(OC(CC[NH2+]C)c2ccccc2)cc1 |
| Flutamide | FC(F)(F)c1cc(NC(=O)C(C)C)ccc1[N+](=O)[O-] |
| Gatifloxacin | Fc1cc2c(N(C=C(C(=O)[O-])C2=O)C2CC2)c(OC)c1N1CC([NH2+]CC1)C |
| Gefitinib | Clc1cc(Nc2ncnc3c2cc(OCCCN2CCOCC2)c(OC)c3)ccc1F |
| Gemfibrozil | O(CCCC(C(=O)[O-])(C)C)c1cc(ccc1C)C |
| Glibenclamide | Clc1cc(C(=O)NCCc2ccc(S(=O)([O-])=NC(=O)NC3CCCCC3)cc2)c(OC)cc1 |
| Glimepiride | S(=O)([O-])(=NC(=O)NC1CCC(CC1)C)c1ccc(cc1)CCNC(=O)N1CC(C)=C(CC)C1=O |
| Haloperidol | Clc1ccc(cc1)C1(O)CC[NH+](CC1)CCCC(=O)c1ccc(F)cc1 |
| Hydrochlorothiazide | Clc1cc2NCNS(=O)(=O)c2cc1S(=O)(=O)N |
| Hydrocodone | O1[C@@H]2[C@]34[C@H]([C@H]([NH+](CC3)C)Cc3c4c1c(OC)cc3)CCC2=O |
| Hydroxyzine | Clc1ccc(cc1)C(N1CC[NH+](CC1)CCOCCO)c1ccccc1 |
| Ibuprofen | O=C([O-])C(C)c1ccc(cc1)CC(C)C |
| Ifosfamide | ClCCN1P(OCCC1)(=O)NCCCl |
| Indomethacin | Clc1ccc(cc1)C(=O)n1c2c(cc(OC)cc2)c(CC(=O)[O-])c1C |
| Irbesartan | O=C1N(Cc2ccc(cc2)-c2ccccc2-c2nn[nH]n2)C(=NC12CCCC2)CCCC |
| Ketoconazole | Clc1cc(Cl)ccc1[C@@]1(O[C@H](CO1)COc1ccc(N2CCN(CC2)C(=O)C)cc1)Cn1ccnc1 |
| Ketoprofen | O=C(c1cc(ccc1)C(C(=O)[O-])C)c1ccccc1 |
| Lansoprazole | S(=O)(Cc1nccc(OCC(F)(F)F)c1C)c1[nH]c2c(n1)cccc2 |
| Lesinurad | Brc1nnc(SCC(=O)[O-])n1-c1c2c(cccc2)c(cc1)C1CC1 |
| Levothyroxine | Ic1cc(cc(I)c1Oc1cc(I)c(O)c(I)c1)C[C@H]([NH3+])C(=O)[O-] |
| Lisinopril | O=C([O-])[C@H]1N(CCC1)C(=O)[C@@H]([NH2+][C@@H](CCc1ccccc1)C(=O)[O-])CCCC[NH3+] |
| Lomefloxacin | Fc1c2N(C=C(C(=O)[O-])C(=O)c2cc(F)c1N1CC([NH2+]CC1)C)CC |
| Loperamide | Clc1ccc(cc1)C1(O)CC[NH+](CC1)CCC(C(=O)N(C)C)(c1ccccc1)c1ccccc1 |
| Loratadine | Clc1cc2c(cc1)\C(\c1ncccc1CC2)=C\1/CCN(CC/1)C(OCC)=O |
| Maprotiline | [NH2+](CCCC12CCC(c3c1cccc3)c1c2cccc1)C |
| Mecillinam | S1[C@H]2N([C@@H](C(=O)[O-])C1(C)C)C(=O)[C@H]2\[NH+]=C\N1CCCCCC1 |
| Mefenamic_acid | O=C([O-])c1ccccc1Nc1cccc(C)c1C |
| Meprobamate | O(CC(CCC)(COC(=O)N)C)C(=O)N |
| Metformin | [NH2+]=C(N(C)C)NC(=[NH2+])N |
| Methadone | O=C(C(CC([NH+](C)C)C)(c1ccccc1)c1ccccc1)CC |
| Metoprolol | O(CC(O)C[NH2+]C(C)C)c1ccc(cc1)CCOC |
| Mianserin | N12C(c3c(Cc4c1cccc4)cccc3)CN(CC2)C |
| Mirtazapine | n1c2N3C(c4c(Cc2ccc1)cccc4)CN(CC3)C |
| Morphine | O1[C@@H]2[C@]34[C@H]([C@H]([NH+](CC3)C)Cc3c4c1c(O)cc3)C=C[C@@H]2O |
| Moxifloxacin | Fc1cc2c(N(C=C(C(=O)[O-])C2=O)C2CC2)c(OC)c1N1C[C@H]2[C@H]([NH2+]CCC2)C1 |
| Naproxen | O(C)c1cc2c(cc(cc2)[C@@H](C(=O)[O-])C)cc1 |
| Nefazodone | Clc1cc(N2CC[NH+](CC2)CCCN2N=C(N(CCOc3ccccc3)C2=O)CC)ccc1 |
| Nordiazepam | Clc1cc2c([NH+]=C([O-])CN=C2c2ccccc2)cc1 |
| Norfloxacin | Fc1cc2c(N(C=C(C(=O)[O-])C2=O)CC)cc1N1CC[NH2+]CC1 |
| Norfluoxetine | FC(F)(F)c1ccc(OC(CC[NH3+])c2ccccc2)cc1 |
| Nortriptyline | [NH2+](CC\C=C/1\c2c(CCc3c\1cccc3)cccc2)C |
| Ofloxacin | Fc1cc2c3N(C=C(C(=O)[O-])C2=O)C(COc3c1N1CCN(CC1)C)C |
| Olaparib | Fc1ccc(cc1C(=O)N1CCN(CC1)C(=O)C1CC1)CC1=NNC(=O)c2c1cccc2 |
| Omeprazole | S(=O)(Cc1ncc(C)c(OC)c1C)c1[nH]c2cc(OC)ccc2n1 |
| Oxazepam | Clc1cc2c(NC(=O)C(O)N=C2c2ccccc2)cc1 |
| Oxycodone | O1[C@@H]2[C@]34CC[NH+]([C@H](Cc5c3c1c(OC)cc5)[C@]4(O)CCC2=O)C |
| Oxytetracycline | OC1=C2[C@@H]([C@](O)(c3c(C2=O)c(O)ccc3)C)[C@H](O)[C@@H]2[C@@]1(O)C(=O)C(C(=O)N)=C([O-])[C@H]2[NH+](C)C |
| Paroxetine | Fc1ccc(cc1)[C@@H]1CC[NH2+]C[C@H]1COc1cc2OCOc2cc1 |
| Pipemidic_acid | O=C1c2c(nc(nc2)N2CC[NH2+]CC2)N(C=C1C(=O)[O-])CC |
| Pizotifen | s1c2c(cc1)/C(/c1c(CC2)cccc1)=C\1/CC[NH+](CC/1)C |
| Primidone | O=C1NCNC(=O)C1(CC)c1ccccc1 |
| Progesterone | O=C1CC[C@@]2([C@@H]3[C@H]([C@@H]4CC[C@H](C(=O)C)[C@]4(CC3)C)CCC2=C1)C |
| Propranolol | O(CC(O)C[NH2+]C(C)C)c1c2c(ccc1)cccc2 |
| Propyphenazone | O=C1N(N(C)C(C)=C1C(C)C)c1ccccc1 |
| Quetiapine | S1c2c(cccc2)C(=Nc2c1cccc2)N1CC[NH+](CC1)CCOCCO |
| Ramipril | O(CC)C(=O)[C@@H](N[C@H](C(=O)N1[C@@H]2[C@H](C[C@H]1C(=O)[O-])CCC2)C)CCc1ccccc1 |
| Repaglinide | O(CC)c1cc(ccc1C(=O)[O-])CC(=O)N[C@@H](CC(C)C)c1ccccc1N1CCCCC1 |
| Risperidone | Fc1cc2onc(c2cc1)C1CC[NH+](CC1)CCC=1C(=O)N2C(=NC=1C)CCCC2 |
| Roxithromycin | O1[C@H](CC)[C@](O)(C)[C@H](O)[C@@H](C)\C(=N\OCOCCOC)\[C@@H](C[C@](O)(C)[C@H](O[C@@H]2O[C@@H](C[C@H]([NH+](C)C)[C@H]2O)C)[C@@H](C)[C@H](O[C@@H]2O[C@@H](C)[C@H](O)[C@](OC)(C2)C)[C@@H](C)C1=O)C |
| Salbutamol | Oc1ccc(cc1CO)C(O)C[NH2+]C(C)(C)C |
| Saxagliptin | OC12CC3(CC(C1)CC(C3)C2)[C@H]([NH3+])C(=O)N1[C@@H]2[C@H](C[C@H]1C#N)C2 |
| Selumetinib | Brc1cc(Cl)c(Nc2c(cc3n(cnc3c2F)C)C(=O)NOCCO)cc1 |
| Sertraline | Clc1cc(ccc1Cl)[C@@H]1CC[C@H]([NH2+]C)c2c1cccc2 |
| Simvastatin | O1[C@@H](C[C@@H](O)CC1=O)CC[C@@H]1[C@@H]2C(=C[C@@H](C[C@@H]2OC(=O)C(CC)(C)C)C)C=C[C@@H]1C |
| Sotalol | S(=O)(=O)(Nc1ccc(cc1)C(O)C[NH2+]C(C)C)C |
| Sparfloxacin | Fc1c(N2C[C@H]([NH2+][C@H](C2)C)C)c(F)c2N(C=C(C(=O)[O-])C(=O)c2c1N)C1CC1 |
| Sulfadiazine | S(=O)([O-])(=Nc1ncccn1)c1ccc(N)cc1 |
| Sulfamethazine | S(=O)([O-])(=Nc1nc(cc(n1)C)C)c1ccc(N)cc1 |
| Sulfamethoxazole | S(=O)([O-])(=Nc1noc(c1)C)c1ccc(N)cc1 |
| Sulfapyridine | S(=O)([O-])(=Nc1ncccc1)c1ccc(N)cc1 |
| Telmisartan | O=C([O-])c1ccccc1-c1ccc(cc1)Cn1c2c(nc1CCC)c(cc(c2)-c1nc2c(n1C)cccc2)C |
| Testosterone | O=C1CC[C@@]2([C@@H]3[C@H]([C@@H]4CC[C@H](O)[C@]4(CC3)C)CCC2=C1)C |
| Ticagrelor | S(CCC)c1nc(N[C@@H]2C[C@H]2c2cc(F)c(F)cc2)c2nnn(c2n1)[C@@H]1C[C@H](OCCO)[C@@H](O)[C@H]1O |
| Tramadol | O(C)c1cc(ccc1)[C@@]1(O)CCCC[C@@H]1C[NH+](C)C |
| Triclocarban | Clc1cc(NC(=O)Nc2ccc(Cl)cc2)ccc1Cl |
| Triclosan | Clc1cc(Cl)ccc1Oc1ccc(Cl)cc1O |
| Trimethoprim | O(C)c1c(OC)cc(cc1OC)Cc1c[nH+]c(nc1N)N |
| Vandetanib | Brc1cc(F)c(Nc2ncnc3c2cc(OC)c(OCC2CC[NH+](CC2)C)c3)cc1 |
| Venlafaxine | O(C)c1ccc(cc1)C(C[NH+](C)C)C1(O)CCCCC1 |
| Verapamil | O(C)c1cc(ccc1OC)C(C(C)C)(CCC[NH+](CCc1cc(OC)c(OC)cc1)C)C#N |
| Zibotentan | S(=O)([O-])(=Nc1ncc(nc1OC)C)c1cccnc1-c1ccc(cc1)-c1ocnn1 |

**1.3 Calculated log *K_ow_* values**

Values for the parent APIs included in the SDF files downloaded from the ChemAxon chemicalize.org website are listed in Table S3. The charge of the species at the experimental pH, or at pH 7.4 when the experimental pH was not provided, was identified by inspecting graphs of concentration versus pH at chemicalize.org.

**Table S3.** Calculated log *K_ow_* values of parent compounds and charge of dominant species at experimental pH, or at pH 7.4 where experimental pH not available. All positively and negatively charged compounds in the dataset had one or two charges of the same sign, except for ceftazidime which had one positive (N^+^) and two negative (O^-^) charges.

| **Compound** | **log *K_ow_*** | **Charge** |
| --- | --- | --- |
| 10,11-Dihydro-10,11-dihydroxy  -carbamazepine | 0.81 | 0 |
| 10,11-Dihydrocarbamazepine | 2.96 | 0 |
| Acetaminophen | 0.91 | 0 |
| Alfuzosin | 1.19 | +1 |
| Allopurinol | 0.03 | 0 |
| Amitriptyline | 4.81 | +1 |
| Amlodipine | 1.64 | +1 |
| Amoxicillin | -2.31 | 0 |
| Androstenedione | 3.93 | 0 |
| Androsterone | 3.77 | 0 |
| Aspirin | 1.24 | -1 |
| Atenolol | 0.43 | 1 |
| Atorvastatin | 5.39 | -1 |
| Atracurium | -0.96 | +2 |
| Azelastine | 4.04 | +1 |
| Azithromycin | 2.44 | 2 |
| Bendroflumethiazide | 1.70 | 0 |
| Bezafibrate | 3.99 | -1 |
| Bicalutamide | 2.71 | 0 |
| Biperiden | 3.54 | +1 |
| Bisoprolol | 2.20 | +1 |
| Bupropion | 3.27 | +1 |
| Candesartan | 5.17 | -2 |
| Carbamazepine | 2.77 | 0 |
| Cediranib | 4.13 | +1 |
| Ceftazidime | -4.55 | -1 |
| Celiprolol | 1.50 | +1 |
| Chlorprothixene | 5.07 | +1 |
| Cimetidine | -0.29 | 0 |
| Ciprofloxacin | -0.81 | 0 |
| Citalopram | 3.76 | +1 |
| Clarithromycin | 3.24 | +1 |
| Clofibric_acid | 2.90 | -1 |
| Clomipramine | 4.88 | +1 |
| Clonazepam | 3.15 | 0 |
| Clotrimazole | 5.84 | 0 |
| Clozapine | 3.40 | +1 |
| Codeine | 1.34 | +1 |
| Cyclophosphamide | 0.10 | 0 |
| Cyproheptadine | 4.38 | +1 |
| Dapagliflozin | 2.11 | 0 |
| Desloratadine | 3.97 | +1 |
| Desmethylmirtazapine | 2.82 | +1 |
| Desmethylsertraline | 4.72 | +1 |
| Desvenlafaxine | 2.29 | +1 |
| Diazepam | 3.08 | 0 |
| Diclofenac | 4.26 | -1 |
| Dicycloverine | 4.93 | +1 |
| Dihydrocodeine | 1.55 | +1 |
| Dilantin | 2.15 | 0 |
| Diphenhydramine | 3.65 | +1 |
| Donepezil | 4.21 | +1 |
| Doxepin | 3.84 | +1 |
| Duloxetine | 4.20 | +1 |
| Enrofloxacin | 1.15 | -1 |
| Ergocalciferol | 7.05 | 0 |
| Erythromycin | 2.60 | +1 |
| Esomeprazole | 2.43 | 0 |
| Estradiol | 3.75 | 0 |
| Estriol | 2.67 | 0 |
| Estrone | 4.31 | 0 |
| Ethinylestradiol | 3.90 | 0 |
| Ezetimibe | 4.56 | 0 |
| Felodipine | 3.44 | 0 |
| Fenoprofen | 3.65 | -1 |
| Fexofenadine | 2.94 | 0 |
| Fleroxacin | 1.12 | -1 |
| Fluoxetine | 4.17 | +1 |
| Flutamide | 3.27 | 0 |
| Gatifloxacin | -0.58 | 0 |
| Gefitinib | 3.75 | 0 |
| Gemfibrozil | 4.39 | -1 |
| Glibenclamide | 3.79 | -1 |
| Glimepiride | 3.12 | -1 |
| Haloperidol | 3.66 | +1 |
| Hydrochlorothiazide | -0.58 | 0 |
| Hydrocodone | 1.96 | +1 |
| Hydroxyzine | 3.41 | +1 |
| Ibuprofen | 3.84 | -1 |
| Ifosfamide | 0.10 | 0 |
| Indomethacin | 3.53 | -1 |
| Irbesartan | 5.50 | -1 |
| Ketoconazole | 4.19 | 0 |
| Ketoprofen | 3.61 | -1 |
| Lansoprazole | 3.03 | 0 |
| Lesinurad | 4.09 | -1 |
| Levothyroxine | 3.73 | 0 |
| Lisinopril | -3.10 | 0 |
| Lomefloxacin | -0.39 | 0 |
| Loperamide | 4.77 | +1 |
| Loratadine | 4.55 | 0 |
| Maprotiline | 4.37 | +1 |
| Mecillinam | -0.55 | 0 |
| Mefenamic_acid | 5.40 | -1 |
| Meprobamate | 0.93 | 0 |
| Metformin | -0.92 | +2 |
| Methadone | 5.01 | +1 |
| Metoprolol | 1.76 | +1 |
| Mianserin | 3.83 | 0 |
| Mirtazapine | 3.21 | 0 |
| Morphine | 0.90 | +1 |
| Moxifloxacin | -0.50 | 0 |
| Naproxen | 2.99 | -1 |
| Nefazodone | 4.65 | 0 |
| Nordiazepam | 3.51 | 0 |
| Norfloxacin | -0.92 | 0 |
| Norfluoxetine | 3.74 | +1 |
| Nortriptyline | 4.43 | +1 |
| Ofloxacin | 0.65 | -1 |
| Olaparib | 1.96 | 0 |
| Omeprazole | 2.43 | 0 |
| Oxazepam | 2.92 | 0 |
| Oxycodone | 1.03 | +1 |
| Oxytetracycline | -4.50 | 0 |
| Paroxetine | 3.15 | +1 |
| Pipemidic acid | -1.77 | 0 |
| Pizotifen | 4.49 | +1 |
| Primidone | 1.12 | 0 |
| Progesterone | 4.15 | 0 |
| Propranolol | 2.58 | +1 |
| Propyphenazone | 2.35 | 0 |
| Quetiapine | 2.81 | +1 |
| Ramipril | 1.47 | -1 |
| Repaglinide | 3.95 | -1 |
| Risperidone | 2.63 | +1 |
| Roxithromycin | 3.00 | +1 |
| Salbutamol | 0.34 | +1 |
| Saxagliptin | -0.08 | +1 |
| Selumetinib | 4.27 | 0 |
| Sertraline | 5.15 | +1 |
| Simvastatin | 4.46 | 0 |
| Sotalol | -0.40 | +1 |
| Sparfloxacin | -0.04 | 0 |
| Sulfadiazine | 0.39 | -1 |
| Sulfamethazine | 0.65 | -1 |
| Sulfamethoxazole | 0.79 | -1 |
| Sulfapyridine | 1.01 | -1 |
| Telmisartan | 6.04 | -1 |
| Testosterone | 3.37 | 0 |
| Ticagrelor | 2.28 | 0 |
| Tramadol | 2.45 | +1 |
| Triclocarban | 4.93 | 0 |
| Triclosan | 4.98 | 0 |
| Trimethoprim | 1.28 | +1 |
| Vandetanib | 4.54 | +1 |
| Venlafaxine | 2.74 | +1 |
| Verapamil | 5.04 | +1 |
| Zibotentan | 1.01 | -1 |

**1.4 MOE descriptors**

The 23 physico-chemical property descriptors calculated by MOE used in the multivariate QSPR models are listed in Table S4.

**Table S4.** MOE molecular descriptors.

| **Descriptor** | **Physico-chemical property** |
| --- | --- |
| Weight | molecular weight |
| b_rotN | number of rotatable bonds |
| Rings | number of rings |
| a_acc | number of hydrogen bond acceptor atoms |
| a_acid | number of acidic atoms |
| a_aro | number of aromatic atoms |
| a_base | number of basic atoms |
| a_don | number of hydrogen bond donor atoms |
| a_hyd | number of hydrophobic atoms |
| vsa_acc | van der Waals surface area of hydrogen bond acceptor atoms |
| vsa_acid | van der Waals surface area of acidic atoms |
| vsa_base | van der Waals surface area of basic atoms |
| vsa_don | van der Waals surface area of hydrogen bond donor atoms |
| vsa_hyd | van der Waals surface area of hydrophobic atoms |
| vsa_pol | van der Waals surface area of polar atoms |
| PC+ | total positive partial charge |
| PC- | total negative partial charge |
| Dens | molecular mass density |
| Dipole | dipole moment |
| Glob | globularity |
| Rgyr | radius of gyration |
| Vol | van der Waals volume |
| VSA | van der Waals surface area |

**1.5 MOE Vsurf descriptors**

The 76 MOE Vsurf descriptors, derived from 3D molecular interaction fields, are listed in Table S5.

**Table S5.** MOE Vsurf descriptors.

| **Vsurf code codecode** | **Property** | **Vsurf code** | **Property** |
| --- | --- | --- | --- |
| V | Interaction field volume | S | Interaction field surface area |
| R | Surface rugosity | G | Surface globularity |
| W | Hydrophilic volume  (8 descriptors) | IW | Hydrophilic integy moment  (8 descriptors) |
| A | Amphiphilic moment | CW | Capacity factor  (8 descriptors) |
| D | Hydrophobic volume  (8 descriptors) | DW | Contact distances of EWmin  (3 descriptors) |
| ID | Hydrophobic integy moment (8 descriptors) | EDmin | Lowest hydrophobic energy  (3 descriptors) |
| CA | Critical packing parameter | Wp | Polar volume  (8 descriptors) |
| HL | Hydrophilic-lipophilic  (2 descriptors) | EWmin | Lowest hydrophilic energy  (3 descriptors) |
| DD | Contact distances of DDmin  (3 descriptors) | HB1 | H-bond donor capacity  (8 descriptors) |

**1.6 Artificial neural networks**

The ANNs used were feed-forward, back-propagation networks with a single hidden layer, as shown in Figure S2. The input layer contains one input node for each descriptor and a single output node for the predicted log *K_d_* value. The number of hidden nodes was varied from 2 to 5.

Input layer

Input 1

Hidden layer

Output layer

Input 2

Input 3

Input 4

Output node

**Figure S1**. Artificial neural network topology.

**2. Results**

**2.1 Univariate linear regression models**

Table S6 shows linear regression models for log *K_d_* against log *K_ow_* and log *D_ow_* at pH 7.4. The APIs with high leverage values reported by Minitab are listed in Table S7. Simple linear regression models for log *K_d_* in terms of log *K_ow_* or log *D_ow_* for the different ion classes with these cases removed are also shown in Table S6.

**Table S6.** Single variable linear regression models for log *K_d_* versus log *K_ow_* or log *D_ow_* for uncharged, positively and negatively charged and zwitterionic APIs, and the complete dataset.

| **Dataset** | **Outliers^a^** | **Model** | ***N*** | ***S*** | ***R^2^*** | ***R^2^_adj_*** | ***R^2^_pred_*** |
| --- | --- | --- | --- | --- | --- | --- | --- |
| Complete dataset |  | log *K_d_* = 2.15 + 0.113 log *K_ow_* | 297 | 1.00 | 0.04 | 0.04 | 0.03 |
|  | 16, 57, 115, 188, 228, 229, 232 | log *K_d_* = 1.90 + 0.190 log *K_ow_* | 290 | 0.97 | 0.10 | 0.09 | 0.08 |
|  |  | log *K_d_* = 2.29 + 0.132 log *D_ow_* | 297 | 0.99 | 0.07 | 0.06 | 0.05 |
|  | 57, 115, 199, 228, 229, 232 | log *K_d_* = 2.22 + 0.162 log *D_ow_* | 291 | 0.97 | 0.09 | 0.08 | 0.07 |
| Uncharged |  | log *K_d_* = 1.07 + 0.424 log *K_ow_* | 92 | 0.67 | 0.47 | 0.47 | 0.45 |
|  | 115, 162, 163, 164 | log *K_d_* = 0.95 + 0.456 log *K_ow_* | 88 | 0.68 | 0.44 | 0.43 | 0.40 |
|  |  | log *K_d_* = 1.94 + 0.329 log *D_ow_* | 92 | 0.74 | 0.36 | 0.35 | 0.33 |
|  | 115 | log *K_d_* = 1.49 + 0.329 log *D_ow_* | 91 | 0.75 | 0.33 | 0.32 | 0.29 |
| Positively charged |  | log *K_d_* = 1.53 + 0.450 log *K_ow_* | 105 | 0.70 | 0.51 | 0.50 | 0.49 |
|  | 31, 199 | log *K_d_* = 1.43 + 0.480 log *K_ow_* | 103 | 0.68 | 0.52 | 0.52 | 0.51 |
|  |  | log *K_d_* = 2.33 + 0.358 log *D_ow_* | 105 | 0.79 | 0.37 | 0.36 | 0.35 |
|  | 199 | log *K_d_* = 2.32 + 0.365 log *D_ow_* | 104 | 0.79 | 0.36 | 0.35 | 0.33 |
| Negatively charged |  | log *K_d_* = 1.87 + 0.003 log *K_ow_* | 76 | 0.87 | 0.01 | 0.00 | 0.00 |
|  | 57 | log *K_d_* = 1.73 + 0.041 log *K_ow_* | 75 | 0.87 | 0.01 | 0.00 | 0.00 |
|  |  | log *K_d_* = 1.87 + 0.018 log *D_ow_* | 76 | 0.87 | 0.00 | 0.00 | 0.00 |
|  | 57, 273 | log *K_d_* = 1.84 + 0.011 log *D_ow_* | 74 | 0.86 | 0.00 | 0.00 | 0.00 |
| Zwitterions |  | log *K_d_* = 3.24 - 0.093 log *K_ow_* | 24 | 0.93 | 0.06 | 0.01 | 0.00 |
|  |  | log *K_d_* = 3.11 - 0.129 log *D_ow_* | 24 | 0.90 | 0.11 | 0.07 | 0.00 |

^a^Cases with high leverage (reported by Minitab) were removed. See Table S1 for compound IDs. N = number of cases, S = standard error, R^2^ = coefficient of determination, R^2^_adj_ = adjusted R^2^, R^2^_pred_ = predicted R^2^.

**Table S7.** High leverage cases removed as outliers from single variable linear regression models, and additional cases removed from multivariate models (*italics*), with their source references.

| **Index** | **Measurement** |
| --- | --- |
| 7 | allopurinol_Berthod (2015) |
| 36 | bendroflumethiazide_Berthod (2015) |
| 16 | amoxicillin_Berthod (2015) |
| 27 | atorvastatin_Stevens-Garmon et al. (2011)_1 |
| 28 | atorvastatin_Stevens-Garmon et al. (2011)_2 |
| 29 | atorvastatin_Yan et al. (2014)_1 |
| 30 | atorvastatin_Yan et al. (2014)_2 |
| 31 | atracurium_Hörsing et al. (2011) |
| 43 | candesartan_AZ |
| 57 | ceftazidime_Berthod (2015) |
| *83* | *dapagliflozin_AZ* |
| *112* | *doxepin_Wick* *et al. (2009)* |
| 115 | ergocalciferol_Berthod (2015) |
| 142 | fexofenadine_Hörsing et al. (2011) |
| 186 | lesinurad_Berthod (2015) |
| 188 | lisinopril_Berthod (2015) |
| 160 | glimepiride_Hörsing et al. (2011) |
| 162 | hydrochlorothiazide_Berthod (2015) |
| 163 | hydrochlorothiazide_Radjenović et al. (2009)_1 |
| 164 | hydrochlorothiazide_Radjenović et al. (2009)_2 |
| 187 | levothyroxine_Berthod (2015) |
| 199 | metformin_Berthod (2015) |
| 200 | methadone_Wick et al. (2009) |
| 228 | oxytetracycline_AZ |
| 229 | oxytetracycline_Berthod (2015) |
| 232 | pipemidic_acid_Jia et al. (2012) |
| *241* | *propyphenazone_Urase & Kikuta (2005)* |
| *244* | *repaglinide_Hörsing et al. (2011)* |
| 248 | roxithromycin_Berthod (2015) |
| 249 | roxithromycin_Yan et al. (2014)_1 |
| 250 | roxithromycin_Yan et al. (2014)_2 |
| 253 | selumetinib_Berthod (2015) |
| *259* | *sotalol_Wick et al. (2009)* |
| 273 | telmisartan_Hörsing et al. (2011) |
| *276* | *ticagrelor_AZ* |
| 280 | triclosan_Hyland et al. (2012) |
| 296 | zibotentan_AZ |
| 297 | zibotentan_Berthod (2015) |

**2.2 Multivariate linear models**

**Table S8**. Number of variables remaining after data reduction protocol.

| **Descriptors** | **Charge class** | **Variables** |
| --- | --- | --- |
| MOE | Uncharged | 19 |
|  | Positive | 21 |
|  | Negative | 18 |
|  | Zwitterions | 15 |
| Vsurf | Uncharged | 39 |
|  | Positive | 43 |
|  | Negative | 27 |
|  | Zwitterions | 15 |
| ParaSurf | Uncharged | 41 |
|  | Positive | 47 |
|  | Negative | 25 |
|  | Zwitterions | 15 |

Tables S9-S11 show the stepwise regression models obtained using the MOE, Vsurf and ParaSurf descriptors for the different charge classes. Results are shown for the complete set of APIs and with high-leverage compounds, identified in Minitab, removed.

**Table S9.** Stepwise regression models for MOE descriptors. Log *D_ow_* = ACD log *D_ow_* at pH 7.4.

| **Dataset** | **Outliers Removed** | **Model** | **N** | **S** | **R^2^** | **R^2^_adj_** | **R^2^_pred_** |
| --- | --- | --- | --- | --- | --- | --- | --- |
| Uncharged |  | log *K_d_* = - 1.15  + 0.46 log *K_ow_* + 2.08 dens  - 0.014 vsa_don | 92 | 0.65 | 0.51 | 0.50 | 0.46 |
|  | 36, 162, 163, 164, 253, 280 | log *K_d_* = 1.63  + 0.69 log *K_ow_*  + 0.10 b_rotN  - 0.033 vsa_don  - 0.12 a_hyd  -0.021 vsa_acc + 0.55 PC+ | 86 | 0.61 | 0.59 | 0.56 | 0.50 |
| Positively charged |  | log *K_d_* = - 3.20  + 0.35 log *K_ow_*  + 0.069 a_aro + 3.21 dens  - 0.30 a_acc - 1.29 glob  - 0.012 vsa_hyd  + 0.016 VSA  - 0.018 vsa_acc | 105 | 0.50 | 0.77 | 0.75 | 0.69 |
|  | 31, 200 | log *K_d_* = - 2.45  + 0.44 log *K_ow_*  - 0.25 a_acc  + 0.059 a_aro + 2.69 dens  - 0.84 glob + 0.015 VSA  - 0.013 vsa_acc  - 0.013 vsa_hyd | 103 | 0.48 | 0.78 | 0.76 | 0.74 |
| Negatively charged |  | log *K_d_* = 1.77 - 0.36 a_acc  - 0.11 a_aro - 7.17 glob  + 0.62 rings  + 0.0052 vsa_hyd | 76 | 0.59 | 0.57 | 0.53 | 0.47 |
|  | 43, 186, 273, 296, 297 | log *K_d_* = 1.44 - 0.40 a_acc  - 0.099 a_aro - 4.66 glob  + 0.77 rings  + 0.0037 vsa_hyd | 71 | 0.57 | 0.55 | 0.51 | 0.46 |
| Zwitterions |  | log *K_d_* = 0.26 - 0.20 log *D_ow_*  + 0.94 rgyr - 0.90 a_base | 24 | 0.79 | 0.39 | 0.30 | 0.17 |
|  | 142 | log *K_d_* = - 1.58 - 0.15 log *D_ow_*  + 1.45 rgyr - 0.93 a_base | 23 | 0.78 | 0.42 | 0.33 | 0.27 |

**Table S10.** Stepwise regression models for Vsurf descriptors.

| **Dataset** | **Outliers Removed** | **Model** | **N** | **S** | **R^2^** | **R^2^_adj_** | **R^2^_pred_** |
| --- | --- | --- | --- | --- | --- | --- | --- |
| Uncharged |  | log *K_d_* = 6.19 + 0.012 D8  + 0.83 EDmin3 + 0.003 Wp1 + 2.71 CP + 0.005 HB1  - 5.40 G | 92 | 0.67 | 0.51 | 0.48 | 0.39 |
|  | 241, 276 | log *K_d_* = 1.83  + 0.46 EWmin1 - 0.0032 D1 + 2.97 CP + 0.006 HB1  - 0.05 DW13 + 0.15 Wp6  + 0.71 ID3 + 0.014 D5  - 0.72 IW2 + 0.059 DD23  + 0.07 IW8 | 90 | 0.59 | 0.64 | 0.59 | 0.48 |
| Positively charged |  | log *K_d_* = 4.315 - 6.9 CW5  - 0.099 DD12 + 0.029 D8  + 0.26 ID8 + 0.10 DD13  + 0.63 W8 - 2.08 R  - 81 CW8 - 0.094 DW13  - 0.093 IW7 + 0.20 ID6  + 0.005 Wp3 | 105 | 0.48 | 0.79 | 0.76 | 0.71 |
|  | 31, 112 | log *K_d_* = - 0.60 - 6.1 CW5  + 0.028 D8 - 0.124 IW7  + 0.269 ID8 + 0.006 Wp3  + 0.074 DD13 + 0.23 ID6  - 0.042 DD12 + 1.14 R | 103 | 0.48 | 0.79 | 0.77 | 0.74 |
| Negatively charged |  | log *K_d_* = 0.028 + 0.52 IW8  - 4.04 IW1 + 42.7 CW7  + 0.15 DW12  + 0.09 DD23 - 1.53 Wp6  + 0.081 Wp4 - 9.6 CW6  + 0.62 CW3 | 76 | 0.57 | 0.61 | 0.57 | 0.46 |
|  | 57, 244 | log *K_d_* = 0.028 + 0.52 IW8  - 4.04 IW1 + 42.7 CW7  + 0.15 DW12  + 0.09 DD23 - 1.53 Wp6  + 0.08 Wp4 - 9.6 CW6  + 0.62 CW3 | 74 | 0.56 | 0.63 | 0.58 | 0.50 |
| Zwitterions* |  | log *K_d_* = 2.71 + 0.016 Wp3 - 2.3 DW13 | 24 | 0.72 | 0.46 | 0.41 | 0.32 |

*No outliers found for zwitterions

**Table S11.** Stepwise regression models for ParaSurf descriptors. See the online manual available at: http://www.ceposinsilico.de/pdf/ParaSurf12.pdf for definitions.

| **Dataset** | **Outliers Removed** | **Model** | **N** | **S** | **R^2^** | **R^2^_adj_** | **R^2^_pred_** |
| --- | --- | --- | --- | --- | --- | --- | --- |
| Uncharged |  | log *K_d_* = - 0.408  + 0.095 polarizability  - 0.62 ENEGskew  + 7.0 FNbal - 0.0008 FN(+)  - 0.02 FNint  + 0.015 ENEGmin  + 0.015 FNmin | 92 | 0.63 | 0.58 | 0.54 | 0.49 |
|  | 83 | log *K_d_* = 5.02  + 0.067 polarizability  + 0.014 MEPvar(+)  - 0.015 ENEGmax  - 0.0041 FNvar(-)  - 0.38 FNrange  - 0.027 FNint  + 0.014 FNmin | 91 | 0.60 | 0.61 | 0.58 | 0.53 |
| Positively charged |  | log *K_d_* = 2.58  - 0.007 FNvar(-)  + 0.41 POLrange  - 4.3 FNbal - 0.068 FNrange + 2.72 EALbalance  + 5.2 POLmin  + 0.17 IELkurt | 105 | 0.60 | 0.66 | 0.64 | 0.58 |
|  | 199, 259 | log *K_d_* = 1.668  - 0.0077 FNvar(-)  + 0.023 POLint  - 0.058 polarizability  - 4.53 FNbal - 6.2 POLvar  - 0.30 HARDkurt  + 0.31 IELkurt  + 0.022 POLkurt  + 2.45 EALbalance  - 0.41 HARDskew | 103 | 0.49 | 0.77 | 0.74 | 0.71 |
| Negatively charged |  | log *K_d_* = - 5.41  + 0.23 FNrange  - 0.61 POLskew  + 66 dipden  + 0.17 EALkurt  + 1.58 IELskew  - 0.019 EALmin  + 0.023 HARDmin  - 0.60 HARDskew | 76 | 0.59 | 0.59 | 0.54 | 0.46 |
|  | 43 | log *K_d_* = -7.09  + 0.23 FNrange  + 56 dipden  - 0.66 POLskew  + 0.17 EALkurt  + 1.70 IELskew  - 0.023 EALmin  + 0.028 HARDmin  - 0.73 HARDskew | 75 | 0.59 | 0.59 | 0.54 | 0.47 |
| Zwitterions* |  | log *K_d_* = 1.86  + 0.009 ENEGmax  - 29 EALbalance  - 0.59 POLskew | 24 | 0.77 | 0.41 | 0.33 | 0.27 |

*No outliers found for zwitterions

Tables S12-S14 show the variables in the PLS models ranked by the absolute values of the loadings on the first three PLS components, averaged over the five choices of test set, for the MOE, Vsurf and ParaSurf descriptors.

**Table S12. MOE descriptors ordered by mean rank over the 5 test sets of the absolute values of the PLS loadings on the first three PLS components.**

| Component 1 | Component 2 | Component 3 |
| --- | --- | --- |
| a_hyd | PC- | dipole |
| vsa_hyd | a_acc | log D*_ow_* |
| VSA | vsa_pol | log *K_ow_* |
| log D*_ow_* | a_don | a_base |
| log *K_ow_* | Weight | rgyr |
| rings | VSA | vsa_pol |
| rgyr | PC+ | vsa_base |
| Weight | vsa_hyd | vsa_acc |
| PC+ | vsa_acc | vsa_don |
| dens | b_rotN | PC- |
| vsa_don | a_hyd | dens |
| a_acid | rgyr | PC+ |
| b_rotN | glob | glob |
| vsa_acc | log *K_ow_* | Weight |
| glob | a_aro | a_acid |
| a_acc | vsa_base | b_rotN |
| vsa_pol | log D*_ow_* | rings |
| a_aro | dens | VSA |
| PC- | a_acid | a_acc |
| a_base | a_base | vsa_hyd |
| dipole | dipole | a_aro |
| vsa_base | vsa_don | a_hyd |
| a_don | rings | a_don |

**Table S13. Vsurf descriptors ordered by mean rank over the 5 test sets of the absolute values of the PLS loadings on the first three PLS components.**

| Component 1 | Component 2 | Component 3 |
| --- | --- | --- |
| vsurf_CW5 | vsurf_Wp2 | vsurf_CW1 |
| vsurf_CW6 | vsurf_G | vsurf_V |
| vsurf_CW4 | vsurf_V | vsurf_R |
| vsurf_HL2 | vsurf_Wp3 | vsurf_D1 |
| vsurf_CW7 | vsurf_D1 | vsurf_HB8 |
| vsurf_EWmin3 | vsurf_HB4 | vsurf_EWmin1 |
| vsurf_EWmin2 | vsurf_HB2 | vsurf_EWmin2 |
| vsurf_EWmin1 | vsurf_HB1 | vsurf_CW2 |
| vsurf_CW3 | vsurf_CW1 | vsurf_EWmin3 |
| vsurf_D6 | vsurf_R | vsurf_HB7 |
| vsurf_D7 | vsurf_CW3 | vsurf_G |
| vsurf_HB7 | vsurf_ID7 | vsurf_D6 |
| vsurf_CW8 | vsurf_ID8 | vsurf_ID4 |
| vsurf_HB4 | vsurf_Wp4 | vsurf_DD12 |
| vsurf_ID5 | vsurf_HB7 | vsurf_CP |
| vsurf_IW8 | vsurf_ID6 | vsurf_CW8 |
| vsurf_HB8 | vsurf_CW4 | vsurf_ID5 |
| vsurf_ID4 | vsurf_D8 | vsurf_HB4 |
| vsurf_D8 | vsurf_D6 | vsurf_ID1 |
| vsurf_ID2 | vsurf_EDmin1 | vsurf_CW3 |
| vsurf_CP | vsurf_EDmin2 | vsurf_ID2 |
| vsurf_D1 | vsurf_EDmin3 | vsurf_A |
| vsurf_ID1 | vsurf_IW3 | vsurf_IW5 |
| vsurf_ID6 | vsurf_HB8 | vsurf_ID6 |
| vsurf_CW2 | vsurf_HL2 | vsurf_CW7 |
| vsurf_HB2 | vsurf_D7 | vsurf_Wp4 |
| vsurf_CW1 | vsurf_CW5 | vsurf_D7 |
| vsurf_IW1 | vsurf_ID2 | vsurf_Wp2 |
| vsurf_IW7 | vsurf_ID5 | vsurf_HB1 |
| vsurf_ID7 | vsurf_ID4 | vsurf_IW4 |
| vsurf_R | vsurf_EWmin1 | vsurf_Wp8 |
| vsurf_IW2 | vsurf_Wp6 | vsurf_DW23 |
| vsurf_HB1 | vsurf_ID1 | vsurf_HB2 |
| vsurf_ID8 | vsurf_EWmin2 | vsurf_Wp7 |
| vsurf_Wp4 | vsurf_IW4 | vsurf_CW4 |
| vsurf_IW4 | vsurf_EWmin3 | vsurf_DD23 |
| vsurf_Wp3 | vsurf_CW6 | vsurf_IW3 |
| vsurf_IW5 | vsurf_Wp7 | vsurf_ID8 |
| vsurf_Wp6 | vsurf_IW2 | vsurf_DW13 |
| vsurf_EDmin1 | vsurf_Wp8 | vsurf_Wp6 |
| vsurf_EDmin3 | vsurf_IW8 | vsurf_IW2 |
| vsurf_IW6 | vsurf_DW13 | vsurf_DD13 |
| vsurf_V | vsurf_IW6 | vsurf_EDmin1 |
| vsurf_EDmin2 | vsurf_IW5 | vsurf_EDmin2 |
| vsurf_Wp7 | vsurf_CW7 | vsurf_IW6 |
| vsurf_DD13 | vsurf_DD12 | vsurf_EDmin3 |
| vsurf_DW12 | vsurf_A | vsurf_DW12 |
| vsurf_Wp8 | vsurf_DW12 | vsurf_IW1 |
| vsurf_DD23 | vsurf_CW8 | vsurf_Wp3 |
| vsurf_Wp2 | vsurf_IW7 | vsurf_CW6 |
| vsurf_DD12 | vsurf_IW1 | vsurf_IW7 |
| vsurf_DW13 | vsurf_DD23 | vsurf_D8 |
| vsurf_A | vsurf_DW23 | vsurf_IW8 |
| vsurf_IW3 | vsurf_CW2 | vsurf_ID7 |
| vsurf_DW23 | vsurf_DD13 | vsurf_HL2 |
| vsurf_G | vsurf_CP | vsurf_CW5 |

**Table S14. ParaSurf descriptors ordered by mean rank over the 5 test sets of the absolute values of the PLS loadings on the first three PLS components.**

| Component 1 | Component 2 | Component 3 |
| --- | --- | --- |
| EALmax | POLrange | FNmean |
| meanMEP+ | POLint | MEP-range |
| MEPvar+ | HARDmin | EALtotvar |
| FNrange | HARDrange | EALrange |
| FNmax | HARDskew | FNvar- |
| EALarea+ | HARDkurt | ENEGbar |
| EALfraction+ | POLvar | MEPvar- |
| MEPskew | POLbar | IELrange |
| FNvar+ | IELkurt | meanMEP- |
| EALbar+ | IELmin | FNabs |
| EALbalance | EALvar+ | dipden |
| FNbal | EALrange | IELvar |
| EALint | EALtotvar | EALskew |
| IELmin | var*balance | FNmin |
| EALvar+ | IELskew | IELmin |
| meanMEP- | MEPbalance | ENEGskew |
| FNskew | POLmin | PS_dipole |
| ENEGkurt | EALbalance | MEPkurt |
| ENEGskew | MEPkurt | IELskew |
| EALkurt | ENEGkurt | FNkurt |
| FNabs | IELrange | EALkurt |
| FNmin | FNmin | POLbar |
| IELvar | EALbar+ | FNskew |
| MEPkurt | HARDvar | HARDvar |
| MEPvar- | FNkurt | HARDmax |
| FNvar- | FNskew | FNrange |
| FNkurt | meanMEP+ | POLvar |
| IELrange | MWt | POLskew |
| IELskew | ENEGbar | POLrange |
| PS_dipole | EALint | POLint |
| volume | ENEGskew | var*balance |
| IELkurt | FNbal | HARDmin |
| polarizability | MEPvar- | globularity |
| globularity | globularity | POLkurt |
| MWt | MEPskew | HARDskew |
| ENEGbar | PS_dipole | FNvar+ |
| POLmin | polarizability | FNmax |
| FNmean | HARDmax | EALint |
| HARDrange | EALfraction+ | HARDrange |
| dipden | MEP-range | MEPskew |
| HARDmax | POLkurt | EALvar+ |
| POLskew | dipden | EALbar+ |
| POLbar | FNrange | MEPbalance |
| var*balance | FNvar- | MEPvar+ |
| POLkurt | EALskew | ENEGkurt |
| HARDkurt | volume | EALmax |
| EALskew | EALarea+ | polarizability |
| EALtotvar | IELvar | volume |
| POLint | MEPvar+ | MWt |
| EALrange | POLskew | HARDkurt |
| HARDvar | EALkurt | FNbal |
| MEP-range | FNmean | IELkurt |
| POLrange | EALmax | EALbalance |
| HARDmin | meanMEP- | EALfraction+ |
| MEPbalance | FNabs | meanMEP+ |
| POLvar | FNvar+ | EALarea+ |
| HARDskew | FNmax | POLmin |

**2.3 Artificial neural networks**

Tables S15-S17 show the results for artificial neural networks for each of the five test sets with 2-5 hidden units using the MOE, Vsurf and ParaSurf descriptors.

**Table S15**. Artificial neural network results for MOE descriptors.

| **Test set** | **Hidden units** | | **MUE_train_** | **MUE_test_** | **R^2^_train_** | **R^2^_test_** |
| --- | --- | --- | --- | --- | --- | --- |
| 1 | 2 | 0.44 | | 0.56 | 0.67 | 0.50 |
| 2 | 2 | 0.45 | | 0.66 | 0.66 | 0.36 |
| 3 | 2 | 0.47 | | 0.55 | 0.64 | 0.56 |
| 4 | 2 | 0.48 | | 0.57 | 0.61 | 0.60 |
| 5 | 2 | 0.47 | | 0.49 | 0.66 | 0.58 |
| mean |  | **0.46** | | **0.57** | **0.65** | **0.52** |
| std. dev. |  | **0.01** | | **0.06** | **0.03** | **0.10** |
| 1 | 3 | 0.39 | | 0.57 | 0.74 | 0.52 |
| 2 | 3 | 0.41 | | 0.66 | 0.74 | 0.28 |
| 3 | 3 | 0.42 | | 0.55 | 0.71 | 0.49 |
| 4 | 3 | 0.41 | | 0.60 | 0.69 | 0.51 |
| 5 | 3 | 0.41 | | 0.58 | 0.73 | 0.12 |
| mean |  | **0.41** | | **0.59** | **0.72** | **0.39** |
| std. dev. |  | **0.01** | | **0.04** | **0.02** | **0.18** |
| 1 | 4 | 0.37 | | 0.57 | 0.75 | 0.53 |
| 2 | 4 | 0.37 | | 0.67 | 0.78 | 0.26 |
| 3 | 4 | 0.39 | | 0.55 | 0.74 | 0.50 |
| 4 | 4 | 0.39 | | 0.60 | 0.73 | 0.52 |
| 5 | 4 | 0.39 | | 0.55 | 0.76 | 0.39 |
| mean |  | **0.38** | | **0.59** | **0.75** | **0.44** |
| std. dev. |  | **0.01** | | **0.05** | **0.02** | **0.12** |
| 1 | 5 | 0.36 | | 0.57 | 0.77 | 0.51 |
| 2 | 5 | 0.35 | | 0.64 | 0.79 | 0.28 |
| 3 | 5 | 0.37 | | 0.53 | 0.77 | 0.52 |
| 4 | 5 | 0.37 | | 0.60 | 0.74 | 0.47 |
| 5 | 5 | 0.37 | | 0.56 | 0.78 | 0.28 |
| mean |  | **0.36** | | **0.58** | **0.77** | **0.41** |
| std. dev. |  | **0.01** | | **0.04** | **0.02** | **0.12** |

MUE = mean unsigned error

**Table S16**. Artificial neural network results for Vsurf descriptors.

| **Test set** | **Hidden units** | **MUE_train_** | **MUE_test_** | **R^2^_train_** | **R^2^_test_** |
| --- | --- | --- | --- | --- | --- |
| 1 | 2 | 0.45 | 0.63 | 0.68 | 0.43 |
| 2 | 2 | 0.44 | 0.71 | 0.69 | 0.26 |
| 3 | 2 | 0.44 | 0.67 | 0.69 | 0.36 |
| 4 | 2 | 0.45 | 0.69 | 0.65 | 0.32 |
| 5 | 2 | 0.47 | 0.56 | 0.65 | 0.50 |
| mean |  | **0.45** | **0.65** | **0.67** | **0.37** |
| std. dev. |  | **0.01** | **0.06** | **0.02** | **0.10** |
| 1 | 3 | 0.39 | 0.68 | 0.74 | 0.21 |
| 2 | 3 | 0.38 | 0.71 | 0.76 | 0.21 |
| 3 | 3 | 0.39 | 0.65 | 0.75 | 0.35 |
| 4 | 3 | 0.39 | 0.66 | 0.73 | 0.36 |
| 5 | 3 | 0.40 | 0.58 | 0.74 | 0.38 |
| mean |  | **0.39** | **0.66** | **0.74** | **0.30** |
| std. dev. |  | **0.01** | **0.05** | **0.01** | **0.09** |
| 1 | 4 | 0.38 | 0.68 | 0.75 | 0.27 |
| 2 | 4 | 0.35 | 0.74 | 0.79 | 0.04 |
| 3 | 4 | 0.36 | 0.67 | 0.77 | 0.31 |
| 4 | 4 | 0.37 | 0.65 | 0.75 | 0.26 |
| 5 | 4 | 0.37 | 0.57 | 0.78 | 0.30 |
| mean |  | **0.37** | **0.66** | **0.77** | **0.24** |
| std. dev. |  | **0.01** | **0.06** | **0.02** | **0.11** |
| 1 | 5 | 0.36 | 0.67 | 0.77 | 0.27 |
| 2 | 5 | 0.34 | 0.72 | 0.81 | 0.13 |
| 3 | 5 | 0.36 | 0.65 | 0.78 | 0.33 |
| 4 | 5 | 0.37 | 0.63 | 0.75 | 0.44 |
| 5 | 5 | 0.35 | 0.57 | 0.79 | 0.36 |
| mean |  | **0.36** | **0.65** | **0.78** | **0.31** |
| std. dev. |  | **0.01** | **0.06** | **0.02** | **0.12** |

MUE = mean unsigned error

**Table S17**. Artificial neural network results for ParaSurf descriptors.

| **Test set** | **Hidden units** | **MUE_train_** | **MUE_test_** | **R^2^_train_** | **R^2^_test_** |
| --- | --- | --- | --- | --- | --- |
| 1 | 2 | 0.42 | 0.58 | 0.71 | 0.50 |
| 2 | 2 | 0.41 | 0.67 | 0.72 | 0.32 |
| 3 | 2 | 0.43 | 0.62 | 0.69 | 0.23 |
| 4 | 2 | 0.41 | 0.66 | 0.69 | 0.41 |
| 5 | 2 | 0.43 | 0.56 | 0.71 | 0.39 |
| mean |  | **0.42** | **0.62** | **0.70** | **0.37** |
| std. dev. |  | **0.01** | **0.05** | **0.01** | **0.10** |
| 1 | 3 | 0.38 | 0.59 | 0.75 | 0.48 |
| 2 | 3 | 0.38 | 0.70 | 0.76 | 0.25 |
| 3 | 3 | 0.39 | 0.64 | 0.75 | 0.23 |
| 4 | 3 | 0.40 | 0.67 | 0.72 | 0.34 |
| 5 | 3 | 0.39 | 0.63 | 0.76 | -0.20 |
| mean |  | **0.39** | **0.64** | **0.75** | **0.22** |
| std. dev. |  | **0.01** | **0.04** | **0.02** | **0.26** |
| 1 | 4 | 0.36 | 0.58 | 0.77 | 0.50 |
| 2 | 4 | 0.36 | 0.70 | 0.78 | 0.15 |
| 3 | 4 | 0.38 | 0.64 | 0.76 | 0.26 |
| 4 | 4 | 0.38 | 0.69 | 0.74 | 0.17 |
| 5 | 4 | 0.37 | 0.57 | 0.78 | 0.41 |
| mean |  | **0.37** | **0.64** | **0.77** | **0.30** |
| std. dev. |  | **0.01** | **0.06** | **0.02** | **0.15** |
| 1 | 5 | 0.36 | 0.57 | 0.77 | 0.53 |
| 2 | 5 | 0.35 | 0.70 | 0.79 | 0.23 |
| 3 | 5 | 0.37 | 0.67 | 0.76 | 0.02 |
| 4 | 5 | 0.37 | 0.66 | 0.75 | 0.24 |
| 5 | 5 | 0.36 | 0.57 | 0.79 | 0.38 |
| mean |  | **0.36** | **0.63** | **0.77** | **0.28** |
| std. dev. |  | **0.01** | **0.06** | **0.02** | **0.19** |

MUE = mean unsigned error

Figures S2-S4 show the dependence of the mean errors on 5 test sets on the number of hidden units, for the MOE, Vsurf and ParaSurf descriptors.

**
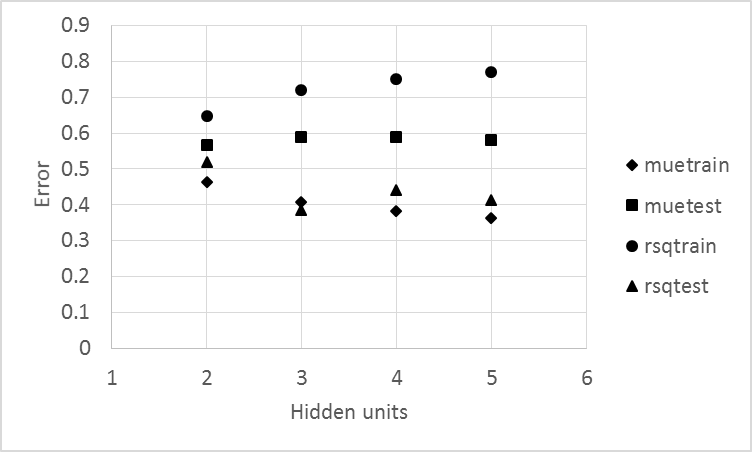
**

**Figure S2**. Artificial neural network results for MOE descriptors, mean errors on 5 test sets versus number of hidden units.


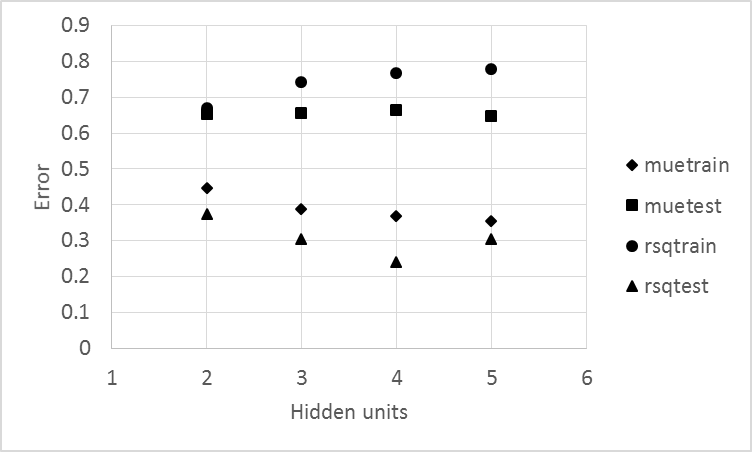


**Figure S3**. Artificial neural network results for Vsurf descriptors, mean errors on 5 test sets versus number of hidden units.


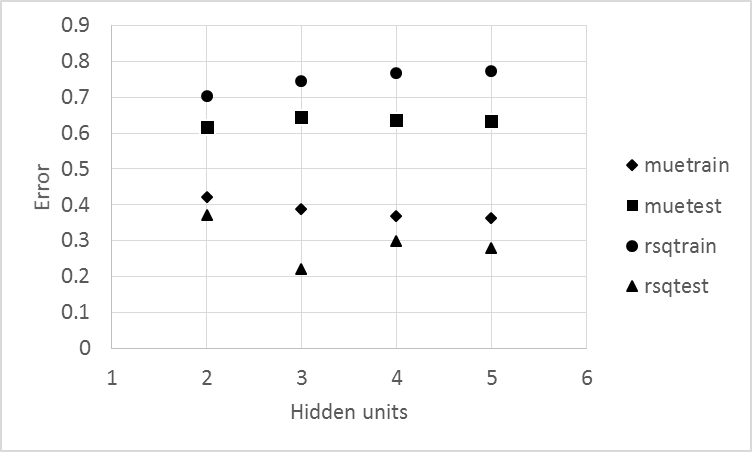


**Figure S4**. Artificial neural network results for ParaSurf descriptors, mean errors on 5 test sets versus number of hidden units.

**Table S18.** Descriptors in artificial neural networks identified by automatic relevance determination, ordered by mean rank over the 5 test sets. See Tables S4 and S5 for definition of MOE and Vsurf descriptors. The ParaSurf descriptors are listed in the online manual available at: http://www.ceposinsilico.de/pdf/ParaSurf12.pdf.

| **MOE** | **Vsurf** | **ParaSurf** |
| --- | --- | --- |
| a_base | vsurf_R | polarizability |
| vsa_hyd | vsurf_G | FNmin |
| PC+ | vsurf_EWmin1 | var*balance |
| a_don | vsurf_CW2 | HARDrange |
| rgyr | vsurf_CW1 | MEPskew |
| vsa_pol | vsurf_HB4 | MEPvar+ |
| log *K_ow_* | vsurf_ID7 | meanMEP- |
| rings | vsurf_IW5 | POLkurt |
| a_acc | vsurf_HL2 | EALkurt |
| Weight | vsurf_ID8 | FNmax |
| VSA | vsurf_DD12 | FNvar+ |
| vsa_base | vsurf_CP | MEPbalance |
| b_rotN | vsurf_ID6 | EALtotvar |
| vsa_don | vsurf_DW13 | EALint |
| dipole | vsurf_HB7 | ENEGskew |
| a_acid | vsurf_Wp6 | EALskew |
| PC- | vsurf_Wp4 | PS_dipole |
| a_aro | vsurf_D1 | EALrange |
| a_hyd | vsurf_IW8 | IELskew |
| vsa_acc | vsurf_CW3 | MEPkurt |
| dens | vsurf_IW4 | FNkurt |
| log *D_ow_* | vsurf_Wp8 | HARDvar |
| glob | vsurf_DD13 | FNabs |
|  | vsurf_CW8 | IELrange |
|  | vsurf_ID4 | IELvar |
|  | vsurf_D8 | EALfraction+ |
|  | vsurf_IW2 | POLrange |
|  | vsurf_EDmin1 | HARDmin |
|  | vsurf_HB1 | HARDskew |
|  | vsurf_EDmin3 | EALbar+ |
|  | vsurf_ID1 | POLmin |
|  | vsurf_IW6 | IELkurt |
|  | vsurf_IW1 | FNmean |
|  | vsurf_IW7 | EALbalance |
|  | vsurf_DW23 | HARDmax |
|  | vsurf_DD23 | globularity |
|  | vsurf_DW12 | EALvar+ |
|  |  | ENEGkurt |
|  |  | HARDkurt |
|  |  | POLvar |
|  |  | FNbal |

Figures S5 and S6 show the performance of PLS and ANN models trained on the whole dataset for the Vsurf and ParaSurf descriptors.


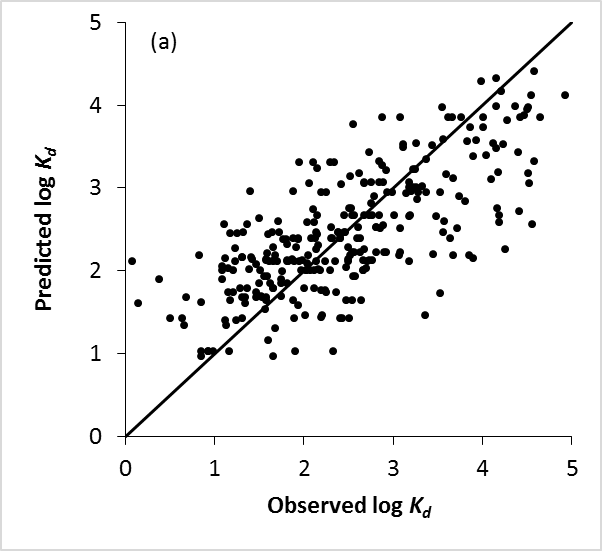

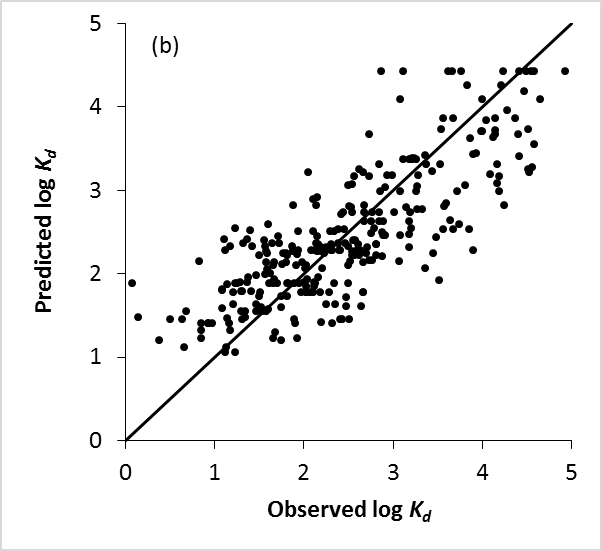


**Figure S5**. Predicted log *K_d_* against observed log *K_d_* for Vsurf descriptors trained on entire dataset for (a) PLS (R^2^ = 0.52, MUE = 0.55), (b) ANN (R^2^ = 0.62, MUE = 0.49). Data point for zibotentan (observed log *K_d_* = -0.699) omitted for clarity.


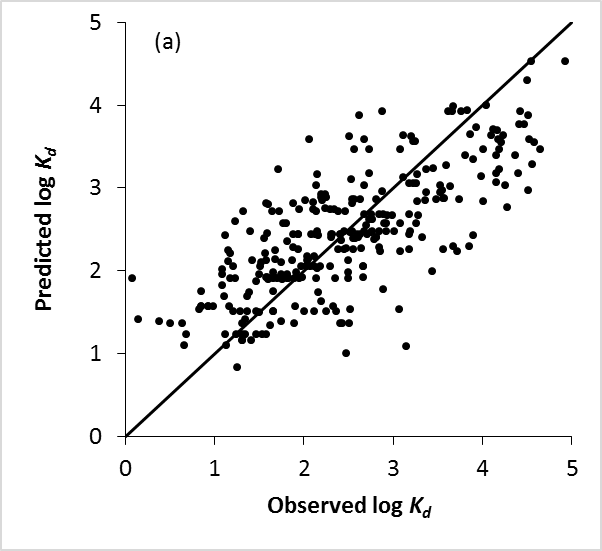

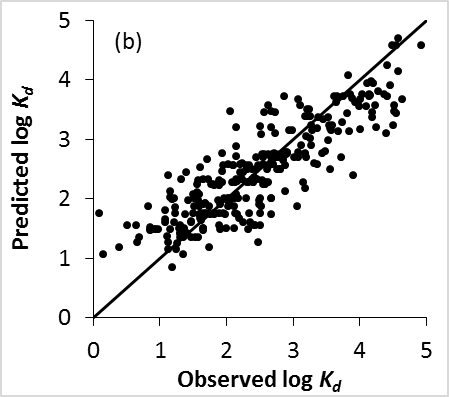


**Figure S6**. Predicted log *K_d_* against observed log *K_d_* for ParaSurf descriptors trained on entire dataset for (a) PLS (R^2^ = 0.56, MUE = 0.54), (b) ANN (R^2^ = 0.69, MUE = 0.44). Data point for zibotentan (observed log *K_d_* = -0.699) omitted for clarity.

**2.4 Predicting log *K_d_* from the artificial neural network model for MOE descriptors**

Algorithm S1 shows the Matlab code to calculate predicted log *K_d_* values for new compounds from the committee of ANNs trained using the MOE descriptors. For those without access to Matlab, the code can also be run in the open source software GNU Octave (http://www.octave.org). The code requires the committee of ANNs, which is provided in Matlab binary format as the Supplementary File *moe_nets.mat*, and a comma-separated data file containing the values of the MOE descriptors. An example input file, containing the MOE descriptors for the compounds on which the committee of networks was trained, is provided as the Supplementary File *moe_training_set.csv*.

**Algorithm S1.** Matlab code to predict log *K_d_* from the committee of ANNs trained using MOE descriptors.

function y = moe_model(CSVFILE)

% Calculate the predicted values of log Kd for the MOE descriptors in

% CSVFILE

% Load the committee of networks

load('moe_nets.mat');

% Load the input data

% Adjust the offsets to match the structure of CSVFILE

% The example file moe_training_set.csv, which contains the data on which

% the committee of networks was trained, contains descriptor names in the

% first row, compound names in the first column and experimental log Kd

% values in the second column.

x = csvread(csvfile,1,2);

nnets = length(nets);

nrows = size(x,1);

% apply the training set standardization

mu = [2.683199, 1.260438, 2.437710, 0.757576, 9.245791, 0.542088, ...

1.047138, 17.181818, 4.508418, 1.044118, 3.476258, 0.140444, ...

2.167431, -2.055747, 3.783813, 2.797980, 353.757347, 23.986719, ...

3.100250, 7.229767, 249.296613, 39.542638, 333.147175];

sigma = [1.884622, 1.975440, 2.463996, 1.160361, 5.196496, 0.770495, ...

1.280493, 6.272576, 3.495281, 0.113060, 2.676474, 0.089706, ...

1.270401, 0.986088, 0.735074, 1.182472, 129.121581, 18.473339, ...

8.762882, 10.608002, 103.606334, 25.912867, 125.371783];

z = (x - repmat(mu,nrows,1)) ./ repmat(sigma,nrows,1);

% apply the networks

a = zeros(nrows,nnets);

for i=1:nnets

v = tanh(z*nets(i).net.w1 + ones(nrows,1)*nets(i).net.b1);

a(:,i) = v*nets(i).net.w2 + ones(nrows,1)*nets(i).net.b2;

end

% average the committee predictions

y = mean(a,2);

**References**

Andersen, H.R., Hansen, M., Kjølholt, J., Stuer-Lauridsen, F., Ternes, T., Halling-Sørensen, B., 2005. Assessment of the importance of sorption for steroid estrogens removal during activated sludge treatment. Chemosphere 61, 139-146.

AstraZeneca. Values taken from internal AstraZeneca reports and AstraZeneca portal https://www.astrazeneca.com/our-company/sustainability.html (accessed 5th May 2016).

Berthod, L., 2015. Mechanistic approach to predicting the sorption characteristics of pharmaceuticals. PhD thesis, University of Portsmouth, UK.

Clara, M., Strenn, B., Saracevic, E., Kreuzinger, N., 2004. Adsorption of bisphenol-A, 17β-estradiole and 17α- ethinylestradiole to sewage sludge. Chemosphere 56, 843-851.

Göbel, A., Thomsen, A., McArdell, C.S., Joss, A., Giger, W., 2005. Environ. Sci. Technol. 39, 3981-3989.

Halling-Sørensen, B., Holten Lützhøft, H.-C., Andersen, H.R., Ingerslev, F., 2000. Environmental risk assessment of antibiotics: comparison of mecillinam, trimethoprim and ciprofloxacin. J. Antimicrob. Chemother. 46, 53-58.

Hörsing, M., Ledin, A., Grabic, R., Fick, J., Tysklind, M., Jansen, J.l.C., Andersen, H.R., 2011. Determination of sorption of seventy-five pharmaceuticals in sewage sludge. Water Res. 45, 4470-4482.

Hyland, K.C., Dickenson, E.R.V., Drewes, J.E., Higgins, C.P., 2012. Sorption of ionized and neutral emerging trace organic compounds onto activated sludge from different wastewater treatment configurations. Water Res. 46, 1958-1968.

Jia, A., Wan, Y., Xiao, Y., Hu, J., 2012. Occurrence and fate of quinolone and fluoroquinolone antibiotics in a municipal sewage treatment plant. Water Res. 46, 387-394.

Lajeunesse, A., Smyth, S., Barclay, K., Sauvé, S., Gagnon, C., 2012. Distribution of antidepressant residues in wastewater and biosolids following different treatment processes by municipal wastewater treatment plants in Canada. Water Res. 46, 5600-5612.

MOE, 2012. Molecular Operating Environment, 2012.10, 1010 Sherbooke St. West, Suite 910, Montreal, QC, Canada, H3A 2R7. www.chemcomp.com

Radjenović, J., Petrović, M., Barceló, D., 2009. Fate and distribution of pharmaceuticals in wastewater and sewage sludge of the conventional activated sludge (CAS) and advanced membrane bioreactor (MBR) treatment. Water Res. 43, 831-841.

Stasinakis, A.S., Thomaidis, N.S., Arvaniti, O.S., Asimakopoulos, A.G., Samaras, V.G., Ajibola, A., Mamais, D., Lekkas, T.D., 2013. Contribution of primary and secondary treatment on the removal of benzothiazoles, benzotriazoles, endocrine disruptors, pharmaceuticals and perfluorinated compounds in a sewage treatment plant. Sci. Total Environ. 463-464, 1067-1075.

Stevens-Garmon, J., Drewes, J.E., Khan, S.J., McDonald, J.A., Dickenson, E.R.V., 2011. Sorption of emerging trace organic compounds onto wastewater sludge solids. Water Res. 45, 3417-3426.

Ternes, T.A., Herrmann, N., Bonerz, M., Knacker, T., Siegrist, H., Joss, A., 2004. A rapid method to measure the solid–water distribution coefficient (K_d_) for pharmaceuticals and musk fragrances in sewage sludge. Water Res. 38, 4075-4084.

Urase, T., Kikuta, T., 2005. Separate estimation of adsorption and degradation of pharmaceutical substances and estrogens in the activated sludge process. Water Res. 39, 1289-1300.

Weininger, D., 1988. SMILES, a chemical language and information system. 1. Introduction to methodology and encoding rules. J. Chem. Inf. Comp. Sci. 28, 31-36.

Weininger, D., Weininger, A., Weininger, J.L., 1989. SMILES. 2. Algorithm for generation of unique SMILES notation. J. Chem. Inf. Comp. Sci. 29, 97-101.

Wick, A., Fink, G., Joss, A., Siegrist, H., Ternes, T.A., 2009. Fate of beta blockers and psycho-active drugs in conventional wastewater treatment. Water Res. 43, 1060-1074.

Yan, Q., Gao, X., Chen, Y-P., Peng, X-Y., Zhang, Y-X., Gan, X-M., Zi, C-F., Guo, J-S., 2014. Occurrence, fate and ecotoxicological assessment of pharmaceutically active compounds in wastewater and sludge from wastewater treatment plants in Chongqing, the Three Gorges Reservoir Area. Sci. Total Environ. 470-471, 618-30.
